# Integrated safety analysis of tofacitinib from Phase 2 and 3 trials of patients with ankylosing spondylitis

**DOI:** 10.1186/s42358-024-00402-x

**Published:** 2024-12-18

**Authors:** Atul Deodhar, Servet Akar, Jeffrey R. Curtis, Bassel El-Zorkany, Marina Magrey, Cunshan Wang, Joseph Wu, Solomon B. Makgoeng, Ivana Vranic, Sujatha Menon, Dona L. Fleishaker, Annette M. Diehl, Lara Fallon, Arne Yndestad, Robert B. M. Landewé

**Affiliations:** 1 Division of Arthritis and Rheumatic Diseases, Oregon Health & Science University, 3181 SW Sam Jackson Park Road, Portland, OR 97239-3098 USA; 2 Faculty of Medicine, Division of Rheumatology, Department of Internal Medicine, Izmir Kâtip Çelebi University, Izmir, Turkey; 3 Division of Clinical Immunology and Rheumatology, University of Alabama at Birmingham, Birmingham, AL USA; 4 Department of Rheumatology, Cairo University, Cairo, Egypt; 5 Division of Rheumatology, Case Western Reserve University, Cleveland, OH USA; 6 Pfizer Inc, Groton, CT USA; 7 Pfizer Inc, Collegeville, PA USA; 8 Pfizer Inc, Tadworth, UK; 9 Pfizer Inc, Montreal, QC Canada; 10 Pfizer Inc, Oslo, Norway; 11 Amsterdam Rheumatology and Immunology Center, Amsterdam University Medical Centers, Amsterdam, The Netherlands; 12 Department of Rheumatology, Zuyderland Medical Center, Heerlen, The Netherlands

**Keywords:** Janus kinase inhibitors/adverse effects, Spondylitis, Ankylosing, Tofacitinib

## Abstract

**Objectives:**

Describe tofacitinib safety from an integrated analysis of randomized controlled trials (RCTs) in patients with ankylosing spondylitis (AS).

**Method:**

Pooled data from Phase 2 (NCT01786668; 04/2013–03/2015)/Phase 3 (NCT03502616; 06/2018–08/2020) RCTs in AS patients were analyzed (3 overlapping cohorts): 16-week placebo-controlled (tofacitinib 5 mg twice daily [BID] [*n* = 185]; placebo [*n* = 187]); 48-week only-tofacitinib 5 mg BID (*n* = 316); 48-week all-tofacitinib (≥ 1 dose of tofacitinib 2, 5, or 10 mg BID; *n* = 420). Baseline 10-year atherosclerotic cardiovascular disease (ASCVD) risk was determined in patients without history of ASCVD (48-week cohorts). Adverse events (AEs)/AEs of special interest were evaluated/compared with findings from other tofacitinib programs (16 Phase 2/Phase 3 rheumatoid arthritis [RA]; 2 Phase 3 psoriatic arthritis [PsA] RCTs) and a real-world cohort of AS patients initiating biologic disease-modifying antirheumatic drugs (US MarketScan).

**Results:**

Most patients (> 75%; 48-week cohorts) without history of ASCVD had low baseline 10-year ASCVD risk. One patient (tofacitinib 5 mg BID; in all 3 cohorts) had a serious infection (aseptic meningitis). Herpes zoster (non-serious) occurred in the 48-week only-tofacitinib 5 mg BID (*n* = 5 [1.6%]) and all-tofacitinib (*n* = 7 [1.7%]; one multi-dermatomal [tofacitinib 10 mg BID]) cohorts. No deaths, opportunistic infections, tuberculosis, malignancies, major adverse cardiovascular events, thromboembolic events, gastrointestinal perforations occurred. Limitations: short RCT durations/low patient numbers within cohorts.

**Conclusion:**

Tofacitinib 5 mg BID was well tolerated to 48 weeks in AS patients; safety profile was consistent with RA/PsA clinical programs and a cohort of AS patients from US routine clinical practice.

**Clinical trial registration numbers:**

NCT01786668 (2013-02-06); NCT03502616 (2018-04-11).

**Supplementary Information:**

The online version contains supplementary material available at 10.1186/s42358-024-00402-x.

## Background

Ankylosing spondylitis (AS), also referred to as radiographic axial spondyloarthritis, is a chronic inflammatory disease [1] associated with back pain, morning stiffness, and spinal rigidity, as well as peripheral and extramusculoskeletal manifestations [2–4]. Risk of cardiovascular (CV) disease is greater in patients with AS vs. the general population [5–7].

Tofacitinib is an oral Janus kinase (JAK) inhibitor for the treatment of AS. The efficacy and safety of tofacitinib in patients with active AS with an inadequate response/intolerance to nonsteroidal anti-inflammatory drugs (NSAIDs) have been established in 16-week Phase 2 [8] and 48-week Phase 3 [9] randomized controlled trials (RCTs).

Two recently published network meta-analyses have compared the efficacy of tofacitinib with other treatments for ankylosing spondylitis. In the first meta-analysis, tofacitinib was the top ranked treatment for ASAS20 response based on the Surface Under the Cumulative Ranking Curve (SUCRA) in AS [10]. In the second meta-analysis, tofacitinib was comparable to tumor necrosis factor inhibitors (TNFi), IL-17 inhibitors, upadacitinib and bimekizumab in the treatment of AS [11]. According to current ASAS-EULAR recommendations, a TNFi, an interleukin-17 inhibitor or a JAK inhibitor such as tofacitinib should be considered if conventional treatment does not sufficiently control disease activity in patients with AS [12]. There are no relevant head-to-head trials in AS, but two systematic literature reviews informing the ASAS-EULAR recommendations indicated similar efficacy versus placebo across these classes of treatment [12–14].

ORAL Surveillance was a safety trial of patients ≥ 50 years of age with rheumatoid arthritis (RA) and CV risk factors, and found increased rates of major adverse cardiovascular events (MACE), malignancies excluding non-melanoma skin cancer, and venous thromboembolism (VTE) with tofacitinib versus TNFi [15]. The mechanism of these safety findings is unknown, and there are no similar prospective studies in AS. However, in line with updated labeling for JAK inhibitors and international guidelines for the management of AS [12], identified differentiating risk factors for the safety outcomes with tofacitinib vs. TNFi (i.e., age ≥ 65 years, long-time current/past smoking, and history of atherosclerotic CV disease [ASCVD; only for MACE]) could inform an individualized approach to treatment decisions [16–18].

We further describe the tofacitinib safety profile in patients with AS, using pooled data from the Phase 2 and 3 RCTs. For context, we present the established safety profile of tofacitinib in RA Phase 2/3 and psoriatic arthritis (PsA) Phase 3 RCTs, along with data from a real-world cohort of patients with AS initiating biologic disease-modifying antirheumatic drugs (bDMARDs).

## Methods

### Study design and patients

Safety data were pooled from 16-week Phase 2 (NCT01786668; 04/2013–03/2015) and 48-week Phase 3 (NCT03502616; 06/2018–08/2020) double-blind, placebo-controlled RCTs [8, 9].

Patients (aged ≥ 18 years) fulfilled modified New York criteria for AS, had active disease, and an inadequate response to ≥ 2 NSAIDs. Exclusion criteria included current (both RCTs) or prior (Phase 2 RCT only) bDMARD use, or targeted synthetic DMARD use [8, 9].

In the Phase 2 RCT, patients received placebo or tofacitinib 2, 5, or 10 mg twice daily (BID) for 12 weeks before a 4-week off-treatment follow-up [8, 9].

### AS Phase 2/3 safety cohorts

**Fig. 1:**
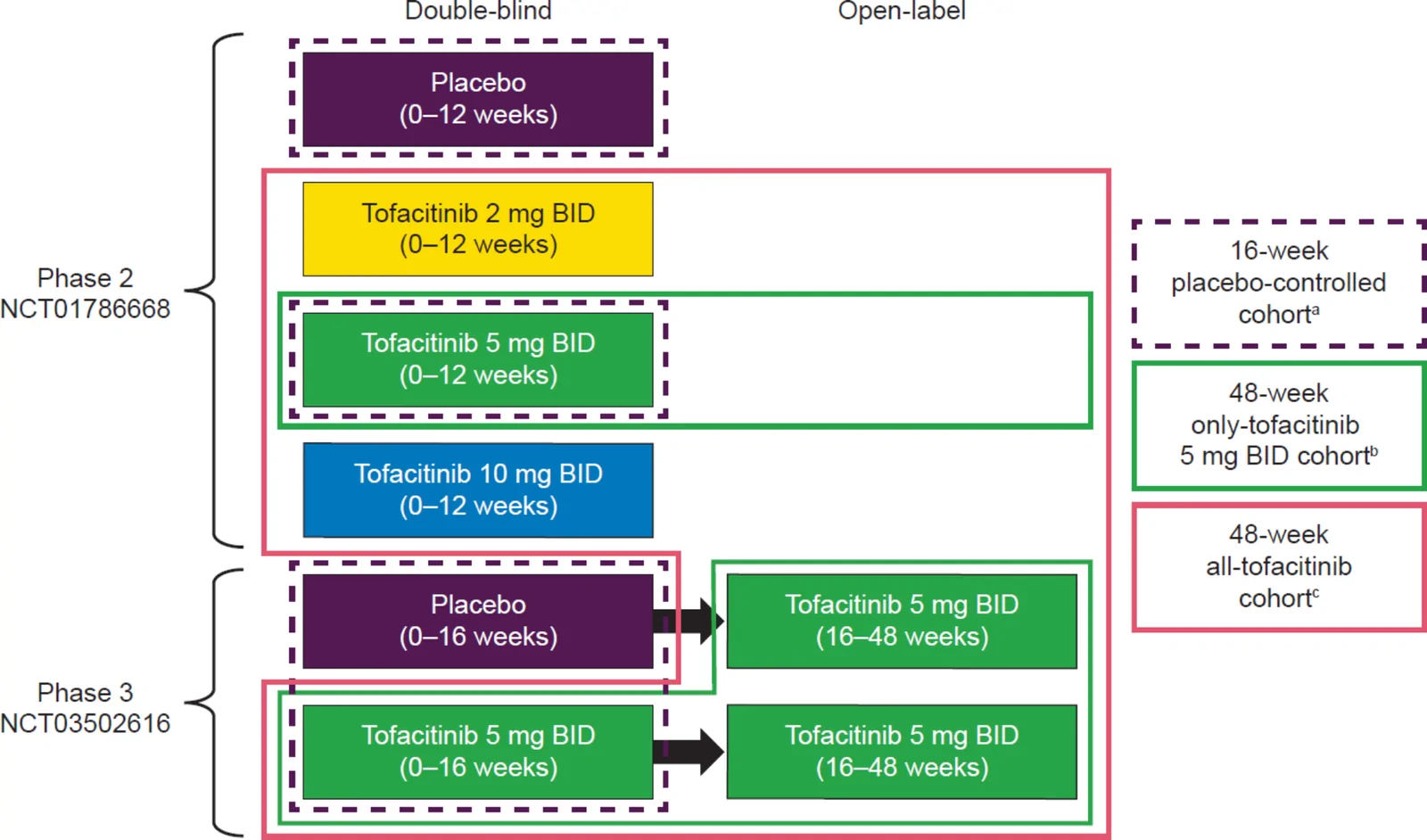
Schematic of the AS Phase 2/3 safety cohorts defined in the current post hoc analysis. ^a^Includes patients who received tofacitinib 5 mg BID or placebo up to week 12 (Phase 2 RCT) or week 16 (Phase 3 RCT). ^b^Includes all patients who received tofacitinib 5 mg BID up to week 12 (Phase 2 RCT) or week 48 (Phase 3 RCT). For patients who switched from placebo to tofacitinib 5 mg BID at week 16 in the Phase 3 RCT, only the open-label tofacitinib-exposed portion was included. ^c^Includes patients who received ≥ 1 dose of tofacitinib 2, 5, or 10 mg BID up to week 12 (Phase 2 RCT) or week 48 (Phase 3 RCT). For patients who switched from placebo to tofacitinib 5 mg BID at week 16 in the Phase 3 RCT, only the open-label tofacitinib-exposed portion was included. *AS* ankylosing spondylitis, *BID* twice daily, *RCT* randomized controlled trial

Three overlapping cohorts were defined: (1) a 16-week placebo-controlled cohort, including patients receiving tofacitinib 5 mg BID or placebo (to week 12 in the Phase 2 RCT and week 16 in the Phase 3 RCT); (2) a 48-week only-tofacitinib 5 mg BID cohort, including all patients receiving tofacitinib 5 mg BID (to week 12 in the Phase 2 RCT and week 48 in the Phase 3 RCT); and (3) a 48-week all-tofacitinib cohort, including patients receiving ≥ 1 dose of tofacitinib 2, 5, or 10 mg BID (to week 12 in the Phase 2 RCT and week 48 in the Phase 3 RCT [Fig. 1]). In the 48-week cohorts, for patients switching from placebo to tofacitinib 5 mg BID at week 16 in the Phase 3 RCT, only the open-label tofacitinib-exposed portion was included.

### Outcomes and analysis

For both 48-week cohorts, baseline 10-year atherosclerotic CV disease (ASCVD) risk was determined post hoc using the ASCVD-pooled cohort equations calculator [19] in patients without history of ASCVD (HxASCVD). Patients were grouped into prespecified categories based on 10-year predicted ASCVD risk: high (≥ 20%); intermediate (≥ 7.5 to < 20%); borderline (≥ 5 to < 7.5%); and low (< 5%).

Safety events included all-treatment-emergent adverse events (TEAEs), serious adverse events (AEs), severe AEs, and discontinuations due to AEs; in the 48-week cohorts, events were assessed overall and by subgroups: age, sex, race, geographical region, prior bDMARDs, and concomitant conventional synthetic DMARDs (csDMARDs). Adjudicated (blinded, independent committees) and nonadjudicated AEs of special interest were evaluated.

Incidence rates (IRs; patients with events/100 patient-years [PY]) and 95% confidence intervals (CIs) were based on a 28-day risk period (first to last study drug + 28 days). For the 16-week placebo-controlled cohort, IRs were calculated via the Cochran–Mantel–Haenszel weighting method adjusting to study with 95% CIs based on large sample approximation. For 48-week cohorts, IRs were estimated without adjusting for study. Exact Poisson (adjusted for PY) 95% CI are provided. Changes from baseline in laboratory and vital signs were examined. Safety analyses were descriptive without formal statistical testing. Missing values were not imputed.

#### Comparisons with RA Phase 2/3 safety cohort, PsA Phase 3 safety cohort, and AS real-world cohort

The RA Phase 2/3 safety cohort included patients randomized to tofacitinib 5 mg BID from 16 completed Phase 2/3 RCTs from the tofacitinib RA clinical program. The PsA Phase 3 safety cohort comprised all patients randomized to tofacitinib 5 mg BID and those switching from placebo to tofacitinib 5 mg BID at week 12 in two completed Phase 3 RCTs of the tofacitinib PsA clinical program (Supplementary Table 1 in Additional file 1).

The AS real-world cohort comprised data from patients aged ≥ 18 years diagnosed with AS (defined as having ≥ 1 inpatient or ≥ 2 outpatient International Classification of Disease diagnosis codes of 720 [AS and other inflammatory spondylopathies] or M45 [AS] on two unique calendar days between January 1, 2010 and December 31, 2017 [≥ 1 code had to be assigned by a rheumatologist]) enrolled in the US MarketScan database. Patients must have had active AS (proxied by use of an anti-AS agent [methotrexate, azathioprine, leflunomide, sulfasalazine, adalimumab, etanercept, infliximab, golimumab, certolizumab pegol, secukinumab, or ixekizumab]) and had to be enrolled in the database for ≥ 12 months before the index date (date of first prescription or administration for AS treatment, or first procedure date after confirmation of AS diagnosis), with no data gap > 30 days. Patient exclusion criteria reflecting those of the tofacitinib AS Phase 3 randomized controlled trial (RCT) [9] were applied where possible. Age- and sex-weighted incidence rates (based on the AS Phase 3 RCT) for safety events are reported for biologic disease-modifying antirheumatic drug (bDMARD) initiators, with data censored at 16 or 48 weeks after first initiation. The AS real-world cohort of bDMARD initiators could have included bDMARD-naïve patients initiating any bDMARD ever or bDMARDexperienced patients initiating a new bDMARD.

## Results

### Patients

#### AS Phase 2/3 safety cohorts

Overall, 372, 316, and 420 patients with AS were in the 16-week placebo-controlled cohort, and 48-week only-tofacitinib 5 mg BID and all-tofacitinib cohorts, respectively. Baseline characteristics were generally similar between tofacitinib 5 mg BID and placebo (16-week placebo-controlled cohort), and consistent with the 48-week all-tofacitinib cohort (Table 1).

**Table 1:** Baseline demographics and disease characteristics^a^ for patients in tofacitinib AS, RA, and PsA safety cohorts

	AS Phase 2/3 safety cohorts^b^				RA Phase 2/3 safety cohort^c^ (*N* = 2664; 2476.7 PY)	PsA Phase 3 safety cohort^d^ (*N* = 347; 196.2 PY)
	16-week placebo-controlled cohort		48-week cohorts			
	Tofacitinib 5 mg BID (*N* = 185; 52.8 PY)	Placebo (*N* = 187; 53.1 PY)	Only-tofacitinib 5 mg BID (*N* = 316; 208.9 PY)	All-tofacitinib (*N* = 420; 233.0 PY)		
Age (years), mean (SD)	41.9 (11.4)	40.5 (11.6)	41.0 (11.3)	41.1 (11.5)	51.8 (12.2)	48.9 (12.0)
≥ 65 years, *n* (%)	7 (3.8)	4 (2.1)	7 (2.2)	13 (3.1)	366 (13.7)	31 (8.9)
Male, *n* (%)	155 (83.8)	140 (74.9)	261 (82.6)	333 (79.3)	460 (17.3)	162 (46.7)
Race, *n* (%)						
White	150 (81.1)	149 (79.7)	252 (79.7)	334 (79.5)	1748 (65.6)	329 (94.8)
Asian	34 (18.4)	38 (20.3)	63 (19.9)	85 (20.2)	98 (3.7)	2 (< 1.0)
Black	0	0	0	0	561 (21.1)	4 (1.2)
Other	1 (0.5)	0	1 (0.3)	1 (0.2)	257 (9.6)	12 (3.5)
Geographic region, *n* (%)^e, f^						
North America	27 (14.6)	15 (8.0)	38 (12.0)	51 (12.1)	566 (21.2)	76 (21.9)
Europe	81 (43.8)	89 (47.6)	136 (43.0)	200 (47.6)	685 (25.7)	229 (66.0)
Asia	32 (17.3)	38 (20.3)	61 (19.3)	83 (19.8)	548 (20.6)	2 (< 1.0)
Latin America	N/A	N/A	N/A	N/A	512 (19.2)	40 (11.5)
Rest of the world	45 (24.3)	45 (24.1)	81 (25.6)	86 (20.5)	353 (13.3)	0
Smoking status, *n* (%)^g^						
Never smoked	95 (51.4)	99 (52.9)	165 (52.2)	217 (51.7)	1759 (66.0)	211 (60.8)
Former smoker	32 (17.3)	24 (12.8)	51 (16.1)	67 (16.0)	449 (16.9)	78 (22.5)
Current smoker	58 (31.4)	64 (34.2)	100 (31.6)	136 (32.4)	395 (14.8)	58 (16.7)
BMI (kg/m^2^), mean (SD) [*N*1]	26.6 (5.5) [184]	26.5 (5.8) [186]	26.4 (5.4) [315]	26.4 (5.3) [419]	27.2 (6.5)	29.9 (6.2)
Disease duration since diagnosis (years), mean (SD)	8.2 (8.6)	6.5 (7.0)	7.5 (8.0)	7.2 (8.3)	7.8 (5.2)	8.4 (7.8)
Extramusculoskeletal manifestations, *n* (%)^f^						
Peripheral arthritis	27 (14.6)	31 (16.6)	50 (15.8)	70 (16.7)	N/A	N/A
Uveitis	34 (18.4)	27 (14.4)	54 (17.1)	73 (17.4)	N/A	2 (0.6)
Psoriasis	7 (3.8)	5 (2.7)	10 (3.2)	12 (2.9)	N/A	95 (27.4)
Inflammatory bowel disease	4 (2.2)	3 (1.6)	6 (1.9)	6 (1.4)	N/A	2 (0.6)
HLA-B27 positive, *n* (%)^h^	161 (87.0)	162 (86.6)	276 (87.3)	369 (87.9)	N/A	N/A
hsCRP (mg/L)						
Mean (SD) [*N*1]	15.4 (16.1)	16.7 (19.1)	16.4 (17.9)	15.2 (17.0)	19.2 (23.8) [2650]	12.0 (20.6)
< 5.0 mg/L, *n* (%)^i^	58 (31.4)	55 (29.4)	91 (28.8)	129 (30.7)	432 (16.3)	126 (36.3)
≥ 5.0 mg/L, *n* (%)^i^	127 (68.6)	132 (70.6)	225 (71.2)	291 (69.3)	2218 (83.7)	221 (63.7)
BASDAI, mean (SD)	6.4 (1.5)	6.5 (1.5)	6.5 (1.5)	6.5 (1.5)	N/A	N/A
BASFI, mean (SD)	5.8 (2.3)	5.8 (2.1)	5.8 (2.2)	5.8 (2.2)	N/A	N/A
PtGA (NRS), mean (SD)	6.8 (1.8)	6.9 (1.7)	6.9 (1.8)	6.8 (1.8)	N/A	N/A
ASDAS (CRP), mean (SD)	3.8 (0.8)	3.8 (0.8)	3.8 (0.8)	3.8 (0.8)	N/A	N/A
Presence of enthesitis based on MASES > 0, *n* (%)	108 (58.4)	114 (61.0)	184 (58.2)	250 (59.5)	N/A	228 (65.7)^j^
MASES^k^, mean (SD) [*N*1]	3.6 (2.6) [108]	3.6 (2.6) [114]	3.6 (2.5) [184]	3.7 (2.6) [250]	N/A	2.8 (1.6)
Presence of swollen joints, *n* (%)	58 (31.4)	54 (28.9)	94 (29.7)	125 (29.8)	N/A	N/A
SJC^l^, mean (SD) [*N*1]	4.7 (6.0) [58]	4.5 (5.6) [54]	4.4 (5.7) [94]	4.7 (5.9) [125]	14.4 (8.7) [2647]	11.9 (9.8)
TJC(68), mean (SD) [*N*1]	N/A	N/A	N/A	N/A	21.6 (14.5) [2647]	20.7 (13.9)
Prior medication, *n* (%)						
2 NSAID-inadequate response	107 (57.8)	94 (50.3)	169 (53.5)	238 (56.7)	N/A	N/A
≥ 3 NSAID-inadequate response	69 (37.3)	89 (47.6)	136 (43.0)	166 (39.5)	N/A	N/A
bDMARD-naïve	154 (83.2)	156 (83.4)	258 (81.6)	362 (86.2)	N/A	155 (44.7)
TNFi-inadequate response or bDMARD use without inadequate response^f^	31 (16.8)	31 (16.6)	58 (18.4)	58 (13.8)	N/A	192 (55.3)^m^
1 TNFi-inadequate response	23 (79.3)	20 (66.7)	41 (74.5)	41 (74.5)	N/A	120 (63.8)^n^
2 TNFi-inadequate response	6 (20.7)	10 (33.3)	14 (25.5)	14 (25.5)	N/A	68 (36.2)^n^
Concomitant medication (day 1), *n* (%)						
NSAIDs	154 (83.2)	156 (83.4)	258 (81.6)	352 (83.8)	N/A	N/A
csDMARD	45 (24.3)	58 (31.0)	89 (28.2)	128 (30.5)	2237 (84.0)^o^	345 (99.4)
Corticosteroids	18 (9.7)	12 (6.4)	25 (7.9)	35 (8.3)	1539 (57.8)	90 (25.9)
Pain management/analgesics, *n* (%)	18 (9.7)	18 (9.6)	28 (8.9)	46 (11.0)	N/A	N/A

*AS* ankylosing spondylitis, *ASDAS(CRP)* Ankylosing Spondylitis Disease Activity Score using high-sensitivity C-reactive protein, *BASDAI* Bath Ankylosing Spondylitis Disease Activity Index, *BASFI* Bath Ankylosing Spondylitis Functional Index, *bDMARD* biologic disease-modifying antirheumatic drug, *BID* twice daily, *BMI* body mass index, *CRP* C-reactive protein, *csDMARD* conventional synthetic disease-modifying antirheumatic drug, *HLA-B27* human leucocyte antigen-B27, *hsCRP* highsensitivity C-reactive protein, *MASES* Maastricht Ankylosing Spondylitis Enthesitis Score, *N* total number of patients included in analysis, *n* number of patients in each analysis category, *N1* number of patients included in the analysis, *N/A* not available, *NRS* numerical rating scale, *NSAID* nonsteroidal anti-inflammatory drug, *PsA* psoriatic arthritis, *PtGA* Patient Global Assessment of Disease Activity, *PY* patient-years, *RA* rheumatoid arthritis, *RCT* randomized controlled trial, *SD* standard deviation, *SJC(44)/(66)* swollen joint count in 44/66 joints, *TJC(68)* tender joint count in 68 joints, *TNFi* tumor necrosis factor inhibitors

^a^In all cohorts, baseline was defined as the last non-missing assessment prior to the first dose of investigational product (including placebo)

^b^Within the 16-week placebo-controlled cohort and 48-week cohorts, patients in the Phase 2 RCT received tofacitinib to week 12

^c^Includes all patients originally randomized to tofacitinib 5 mg BID from 16 completed Phase 2/3 RCTs from the tofacitinib RA clinical program

^d^Includes all patients randomized to tofacitinib 5 mg BID and those who switched from placebo to tofacitinib 5 mg BID at week 12 in the two completed Phase 3 RCTs of the tofacitinib PsA clinical program

^e^In the AS Phase 2/3 safety cohorts, North America includes Canada and the US; Europe includes Bulgaria, Czech Republic, France, Hungary, Poland, and Spain; Asia includes China, South Korea, and Taiwan; and rest of the world includes Australia, Russia, Turkey, and Ukraine. In the RA Phase 2/3 safety cohort: Europe includes Austria, Belgium, Bulgaria, Croatia, Czech Republic, Denmark, Estonia, Finland, France, Germany, Greece, Hungary, Ireland, Italy, Latvia, Lithuania, Poland, Romania, Slovakia, Spain, Sweden, and UK; Asia includes China, India, Japan, Republic of Korea, Malaysia, Philippines, Taiwan, and Thailand; Latin America includes Argentina, Brazil, Chile, Colombia, Cost Rica, Dominican Republic, Mexico, Peru, Puerto Rico, and Venezuela; and rest of the world includes Australia, Bosnia and Herzegovina, Israel, New Zealand, Russia, South Africa, Turkey, and Ukraine. In the PsA Phase 3 safety cohort: Europe includes Bulgaria, Czech Republic, Hungary, Poland, Slovakia, Belgium, France, Germany, Spain, UK, Australia, and Russia; Asia includes Taiwan; and Latin America includes Mexico and Brazil

^f^Percentages based on *N* and includes patients with missing data

^g^RA Phase 2/3 safety cohort: missing data for 61 patients

^h^If baseline results were not available, results after baseline are included

^i^Thresholds of ≤ 0.3 and > 0.3 mg/dL in the RA Phase 2/3 safety cohort, and ≤ 2.87 and > 2.87 mg/L in the PsA Phase 3 safety cohort

^j^Measured by Leeds Enthesitis Index > 0 in the PsA Phase 3 safety cohort

^k^In patients with MASES > 0 in the AS Phase 2/3 safety cohorts; in patients with Leeds Enthesitis Index > 0 in the PsA Phase 3 safety cohort

^l^In patients with SJC(44) > 0 for AS, and SJC(66) ≥ 0 for RA and PsA

^m^Prior bDMARD-experienced in the PsA Phase 3 safety cohort

^n^Percentages are based on patients with known inadequate response to bDMARD (TNFi or non-TNFi) in the PsA Phase 2/3 safety cohort

^o^Methotrexate use for the RA Phase 2/3 safety cohort

**Fig. 2:**
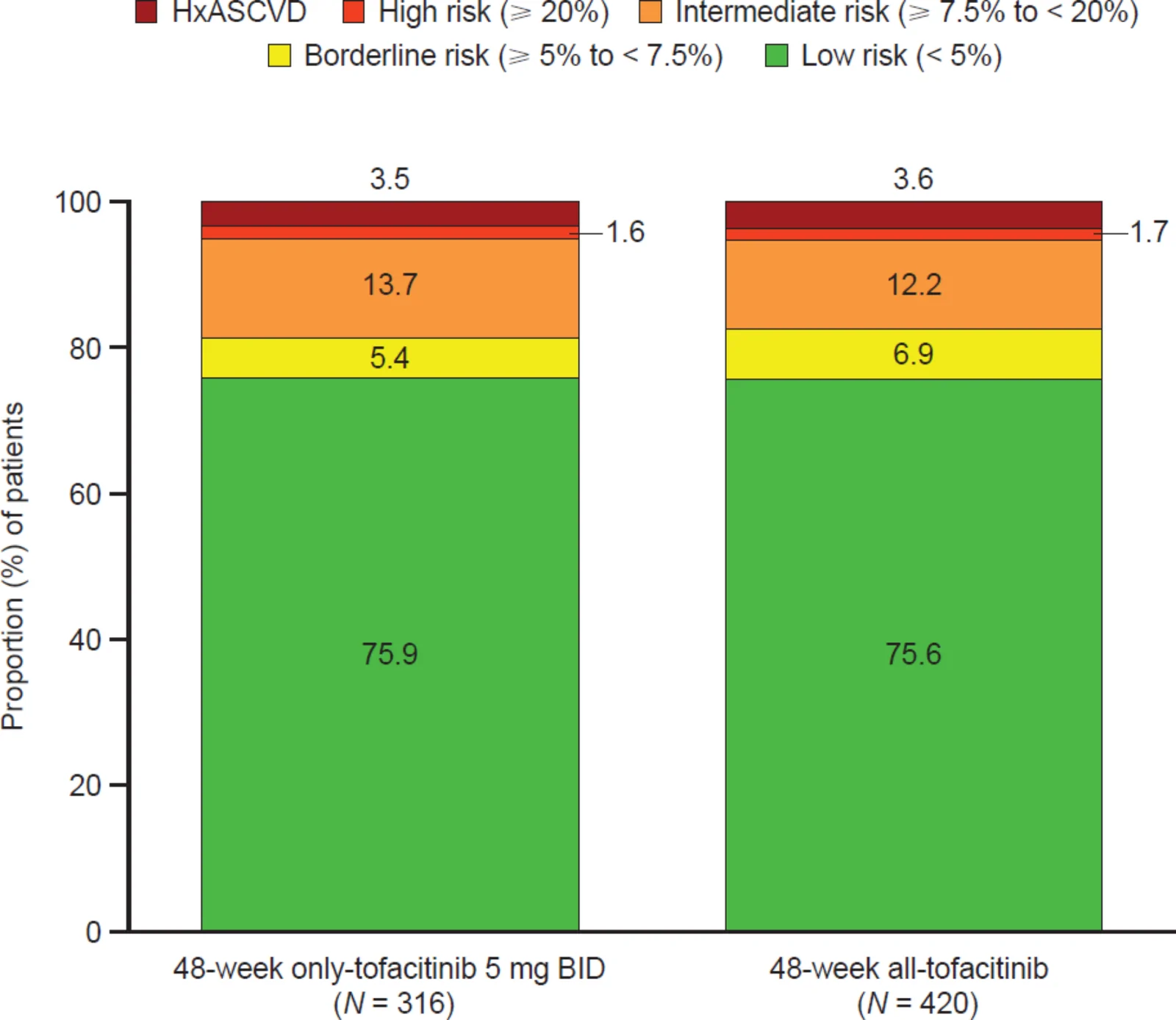
Baseline CV risk profile of patients in the tofacitinib AS Phase 2/3 safety cohorts. HxASCVD was defined as ≥ 1 occurrence of CAD, CeVD, or PAD. History of CAD, CeVD, or PAD was identified in patients’ general medical history through MedDRA Preferred Terms consistent with one of these conditions and reflecting prior/ongoing events, procedures, or diagnoses. Percentages were based on all patients as the denominator (*n* = 316 for 48-week only-tofacitinib 5 mg BID cohort; *n* = 420 for 48-week all-tofacitinib cohort). Baseline 10-year ASCVD risk was determined using the ASCVD-PCE risk calculator, developed by the American College of Cardiology/American Heart Association [19]. Scores were calculated based on patients’ age, sex, race (White, Black, other), smoking status (yes/no), systolic blood pressure, antihypertensive treatment (yes/no), total cholesterol, high-density lipoprotein cholesterol, and diabetes (yes/no). Percentages based on all patients with nonmissing values. Patients without HxASCVD but with missing ASCVD-PCE risk data: 48-week only-tofacitinib 5 mg BID cohort, *n* = 1; 48-week all-tofacitinib cohort, *n* = 2. *AS* ankylosing spondylitis, *ASCVD* atherosclerotic cardiovascular disease, *BID* twice daily, *CAD* coronary artery disease, *CeVD* cerebrovascular disease, *Hx* history (of), *MedDRA* Medical Dictionary for Regulatory Activities, *N* total number of patients included in analysis, *n* number of patients with characteristic, *PAD* peripheral artery disease, *PCE* pooled cohort equations, *RCT* randomized controlled trial

At baseline, 3.5% and 3.6% of patients in the 48-week only-tofacitinib 5 mg BID and all-tofacitinib cohorts, respectively, had HxASCVD; in patients without HxASCVD, > 75% had low (< 5%) baseline 10-year ASCVD risk (Fig. 2; Supplementary Table 2 in Additional File 2).

#### RA Phase 2/3 and PsA Phase 3 safety cohorts

The RA Phase 2/3 and PsA Phase 3 safety cohorts included 2664 and 347 patients, respectively; at baseline, patients were older vs. the AS Phase 2/3 safety cohorts, and included greater proportions of concomitant csDMARD and oral corticosteroid users, and fewer males and current smokers (Table 1).

#### AS real-world cohort

The AS real-world cohort comprised 2253 patients; 39.5% had received prior bDMARDs (Supplementary Table 3 in Additional File 2). At baseline, vs. the AS Phase 2/3 safety cohorts, there was a greater proportion of females and patients with prior oral corticosteroid use.

### Overview of AEs in the AS Phase 2/3 safety cohorts

In the 16-week placebo-controlled cohort, proportions of patients with TEAEs, serious AEs, severe AEs, and discontinuations due to AEs were similar between tofacitinib 5 mg BID and placebo (Table 2). Uveitis was reported in one (0.5%) and three (1.6%) patients with history of uveitis receiving tofacitinib 5 mg BID and placebo, respectively. Psoriasis occurred in one (0.5%) patient (placebo) with history of psoriasis.

**Table 2:** Summary of AEs in the tofacitinib AS phase 2/3 safety cohorts^a^

	16-week placebo-controlled cohort		48-week cohorts	
	Tofacitinib 5 mg BID (*N* = 185)	Placebo (*N* = 187)	Only-tofacitinib 5 mg BID (*N* = 316)	All-tofacitinib (*N* = 420)
General AEs, *n* (%)				
All TEAEs	101 (54.6)	92 (49.2)	201 (63.6)	251 (59.8)
Serious AEs^b^	3 (1.6)	2 (1.1)	10 (3.2)	11 (2.6)
Severe AEs	3 (1.6)	3 (1.6)	7 (2.2)	8 (1.9)
Discontinued from study due to AEs	1 (0.5)	3 (1.6)	2 (0.6)	3 (0.7)
Discontinued from study drug due to AEs	4 (2.2)	4 (2.1)	11 (3.5)	12 (2.9)
Dose reduction/temporary discontinuation due to AE	12 (6.5)	6 (3.2)	30 (9.5)	32 (7.6)
Deaths	0	0	0	0
TEAEs (all causalities) in ≥ 2% of patients by Preferred Term, *n* (%)				
Upper respiratory tract infection	14 (7.6)	11 (5.9)	32 (10.1)	39 (9.3)
Nasopharyngitis	13 (7.0)	13 (7.0)	25 (7.9)	31 (7.4)
Diarrhea	7 (3.8)	6 (3.2)	14 (4.4)	16 (3.8)
ALT increased	6 (3.2)	1 (0.5)	11 (3.5)	12 (2.9)
Influenza	6 (3.2)	1 (0.5)	9 (2.8)	9 (2.1)
Urine protein present	5 (2.7)	2 (1.1)	11 (3.5)	11 (2.6)
Arthritis	4 (2.2)	1 (0.5)	5 (1.6)	5 (1.2)
AST increased	4 (2.2)	0	7 (2.2)	7 (1.7)
Fatigue	4 (2.2)	1 (0.5)	9 (2.8)	10 (2.4)
Headache	4 (2.2)	4 (2.1)	11 (3.5)	15 (3.6)
Respiratory tract infection viral	4 (2.2)	0	5 (1.6)	5 (1.2)
Arthralgia	3 (1.6)	8 (4.3)	7 (2.2)	8 (1.9)
Blood creatine phosphokinase increased	3 (1.6)	2 (1.1)	8 (2.5)	9 (2.1)
Cough	3 (1.6)	3 (1.6)	7 (2.2)	9 (2.1)
Oropharyngeal pain	3 (1.6)	1 (0.5)	8 (2.5)	9 (2.1)
Dizziness	1 (0.5)	4 (2.1)	3 (0.9)	3 (0.7)
Spinal pain	1 (0.5)	4 (2.1)	2 (0.6)	4 (1.0)
Weight increased	1 (0.5)	1 (0.5)	10 (3.2)	10 (2.4)
Abdominal pain upper	0	5 (2.7)	5 (1.6)	10 (2.4)
Hepatic function abnormal	0	0	8 (2.5)	9 (2.1)

MedDRA version 23.0 coding dictionary applied. TEAE definition in the Phase 2 RCT: on-treatment events that are new or worsened in severity relative to the pretreatment period prior to day 1. TEAE definition in the Phase 3 RCT: on-treatment events that start during the effective duration of treatment

*AE* adverse event, *ALT* alanine aminotransferase, *AS* ankylosing spondylitis, *AST* aspartate aminotransferase, *BID* twice daily, *MedDRA* Medical Dictionary for Regulatory Activities, *N* total number of patients included in analysis, *n* number of patients with events, *RCT* randomized controlled trial, *TEAE* treatment-emergent adverse event

^a^Within the 16-week placebo-controlled and 48-week cohorts, patients in the Phase 2 RCT received tofacitinib to week 12

^b^For serious AEs, data presented are based on the clinical database. One patient in the placebo arm who switched to tofacitinib 5 mg BID in the Phase 3 RCT had a serious AE with onset at day 160 (i.e., after switching to tofacitinib) in the clinical database but at day 55 (before switching to tofacitinib) in the Sponsor’s Safety Data Warehouse. This patient was included in the 48-week cohorts rather than the 16-week placebo-controlled cohort

In the 48-week cohorts, < 4% of patients reportedserious AEs, severe AEs, and discontinuations due to AEs (Table 2). Four (1.3%) and six (1.4%) patients in the 48-week only-tofacitinib 5 mg BID and all-tofacitinib cohorts, respectively, experienced uveitis; all but one patient (tofacitinib 2 mg BID) had history of uveitis.

No TEAEs of inflammatory bowel disease occurred; most TEAEs were mild/moderate. No deaths were reported.

Safety event IRs by age, sex, race, geographic region, prior bDMARD history, and Day 1 concomitant csDMARD use, in the 48-week cohorts are shown in Supplementary Tables 4–9 in Additional File 3 (noting the small numbers of patients in some subgroups and overlapping CIs for some comparisons).

### AEs of special interest in the AS Phase 2/3 safety cohorts, RA Phase 2/3 and PsA Phase 3 safety cohorts, and AS real-world cohort

#### Serious infections

One patient receiving tofacitinib 5 mg BID (included in all AS Phase 2/3 safety cohorts) had a serious infection (aseptic meningitis [Table 3]). No serious infections were reported with placebo.

**Table 3:** General AEs and AEs of special interest in tofacitinib AS, RA, and PsA safety cohorts

	AS Phase 2/3 safety cohorts^a^				RA Phase 2/3 safety cohort^b^ (*N* = 2664; 2476.7 PY)	PsA Phase 3 safety cohort^c^ (*N* = 347; 196.2 PY)
	16-week placebo-controlled cohort		48-week cohorts			
	Tofacitinib 5 mg BID (*N* = 185; 52.8 PY)	Placebo (*N* = 187; 53.1 PY)	Only-tofacitinib 5 mg BID (*N* = 316; 208.9 PY)	All-tofacitinib (*N* = 420; 233.0 PY)		
*n (%)* *IR [95% CI*,* events per 100 PY]*						
General AEs						
Serious AEs	3 (1.6) 5.28 [0–11.25]	2 (1.1) 3.56 [0–8.49]	8 (2.5) 3.49 [1.51–6.87]	9 (2.1) 3.45 [1.58–6.55]	242 (9.1) 9.73 [8.54–11.03]	15 (4.3) 7.57 [4.24–12.49]
Discontinuation of study treatment due to AEs	4 (2.2) 7.04 [0.14–13.94]	4 (2.1) 7.10 [0.14–14.05]	11 (3.5) 4.77 [2.38–8.54]	12 (2.9) 4.58 [2.37–8.00]	N/A	N/A
All-cause mortality	0 0 [0–3.28]	0 0 [0–3.31]	0 0 [0–1.59]	0 0 [0–1.40]	8 (0.3) 0.31 [0.13–0.61]	1 (0.3) 0.50 [0.01–2.77]
AEs of special interest						
Serious infections^d^	1 (0.5) 1.77 [0–5.89]	0 0 [0–3.31]	1 (0.3) 0.43 [0.01–2.41]	1 (0.2) 0.38 [0.01–2.12]	67 (2.5) 2.61 [2.02–3.31]	4 (1.2) 1.99 [0.54–5.10]
Opportunistic infections (excluding tuberculosis)^e^	0 00 [0–3.28]	0 0 [0–3.31]	0 0 [0–1.59]	0 0 [0–1.40]	9 (0.3) 0.35 [0.16–0.66]	1 (0.3) 0.50 [0.01–2.77]
Tuberculosis^e^	0 0 [0–3.28]	0 0 [0–3.31]	0 0 [0–1.59]	0 0 [0–1.40]	2 (0.1) 0.08 [0.01–0.28]	0 0 [0–1.83]
Herpes zoster	0 0 [0–3.28]	0 0 [0–3.31]	5 (1.6) 2.18 [0.71–5.08]	7 (1.7) 2.68 [1.08–5.53]	74 (2.8) 2.92 [2.29–3.66]	3 (0.9) 1.50 [0.31–4.39]
Malignancies, excluding NMSC^e^	0 0 [0–3.28]	0 0 [0–3.31]	0 0 [0–1.59]	0 0 [0–1.40]	9 (0.3) 0.35 [0.16–0.66]	3 (0.9) 1.49 [0.31–4.37]
NMSC^e^	0 0 [0–3.28]	0 0 [0–3.31]	0 0 [0–1.59]	0 0 [0–1.40]	11 (0.4) 0.43 [0.21–0.76]	0 0 [0–1.83]
MACE^e, f^	0 0 [0–3.28]	0 0 [0–3.31]	0 0 [0–1.59]	0 0 [0–1.40]	7 (0.3) 0.28 [0.11–0.58]	1 (0.3) 0.50 [0.01–2.77]
VTE^e, g^	0 0 [0–3.28]	0 0 [0–3.31]	0 0 [0–1.59]	0 0 [0–1.40]	7 (0.3) 0.27 [0.11–0.56]	0 0 [0–1.83]
DVT^e^	0 0 [0–3.28]	0 0 [0–3.31]	0 0 [0–1.59]	0 0 [0–1.40]	4 (0.2) 0.15 [0.04–0.40]	0 0 [0–1.83]
PE^e^	0 0 [ 0–3.28]	0 0 [ 0–3.31]	0 0 [0–1.59]	0 0 [0–1.40]	3 (0.1) 0.12 [0.02–0.34]	0 0 [0–1.83]
ATE^e^	0 0 [0–3.28]	0 0 [0–3.31]	0 0 [0–1.59]	0 0 [0–1.40]	6 (0.2) 0.23 [0.09–0.51]	1 (0.3) 0.50 [0.01–2.78]
GI perforation^e^	0 0 [0–3.28]	0 0 [0–3.31]	0 0 [0–1.59]	0 0 [0–1.40]	0 0 [0–0.14]	1 (0.3) 0.50 [0.01–2.77]
Interstitial lung disease^e^	0 0 [0–3.28]	0 0 [0–3.31]	0 0 [0–1.59]	0 0 [0–1.40]	3 (0.1) 0.12 [0.02–0.34]	0 0 [0–1.83]
Hepatic steatosis	2 (1.1) 3.54 [0–8.94]	0 0 [0–3.31]	4 (1.3) 1.74 [0.47–4.45]	5 (1.2) 1.91 [0.62–4.46]	N/A	N/A
Transaminase elevations	8 (4.3) 14.27 [4.38–24.16]	2 (1.1) 3.55 [0–8.47]	24 (7.6) 10.92 [7.00–16.25]	28 (6.7) 11.18 [7.43–16.15]	N/A	N/A

MedDRA version 23.0 coding dictionary applied. IRs (patients with events/100 PY) and 95% CIs based on a 28-day risk period (time of first to last study drug dose + 28 days). For patients who switched from placebo to tofacitinib 5 mg BID after week 16, the first dose date refers to the date of the first tofacitinib dose. IRs for the 16-week placebo-controlled cohort were based on Cochran–Mantel–Haenszel weighting method adjusting to study with 95% CIs based on large sample approximation. For the 48-week cohorts, IRs were estimated without adjusting for study. Exact Poisson (adjusted for PY) 95% CIs are provided for IRs

*AE* adverse event, *AS* ankylosing spondylitis, *ATE* arterial thromboembolism, *BID* twice daily, *CI* confidence interval, *DVT* deep vein thrombosis, *GI* gastrointestinal, *IR* incidence rate, *MACE* major adverse cardiovascular events, *MedDRA* Medical Dictionary for Regulatory Activities, *N* total number of patients included in analysis, *n* number of patients with event, *N/A* not available, *NMSC* nonmelanoma skin cancer, *PE* pulmonary embolism, *PsA* psoriatic arthritis, *PY* patient-years, *RA* rheumatoid arthritis, *RCT* randomized controlled trial, *VTE* venous thromboembolism

^a^Within the 16-week placebo-controlled and 48-week cohorts, patients in the Phase 2 RCT received tofacitinib to week 12

^b^Includes all patients randomized to tofacitinib 5 mg BID from 16 completed Phase 2/3 RCTs from the tofacitinib RA clinical program

^c^Includes all patients randomized to tofacitinib 5 mg BID and those who switched from placebo to tofacitinib 5 mg BID in the 2 completed Phase 3 RCTs of the tofacitinib PsA clinical program

^d^Any event in the Infections and Infestations System Organ Class and classified as serious

^e^Adjudicated events in both AS Phase 2 and 3 RCTs

^f^Includes nonfatal myocardial infarction, nonfatal stroke, or death from cardiovascular causes

^g^Includes DVT and/or PE

Serious infection IRs (95% CIs) were 1.77 (0–5.89) vs. 0 (0–3.31) per 100 PY for the tofacitinib 5 mg BID vs. placebo arms (16-week placebo-controlled cohort). Serious infection IRs (95% CIs) were 0.43 (0.01–2.41) and 0.38 (0.01–2.12) per 100 PY for the 48-week only-tofacitinib 5 mg BID and all-tofacitinib cohorts, respectively; these were numerically lower vs. the RA Phase 2/3 and PsA Phase 3 safety cohorts (Table 3), and the AS real-world cohort (Supplementary Table 10 in Additional File 4), with 95% CIs mostly overlapping.

#### Opportunistic infections

No opportunistic infections were reported in the AS Phase 2/3 safety cohorts (Table 3); three events were assessed by an independent adjudication committee (aseptic meningitis and ophthalmic herpes simplex [tofacitinib 5 mg BID]; herpes zoster [tofacitinib 10 mg BID]), but did not meet opportunistic infection criteria. Across other cohorts, opportunistic infections IRs were ≤ 0.5 per 100 PY, with overlapping 95% CIs (Table 3; Supplementary Table 10 in Additional File 4).

#### Tuberculosis

No tuberculosis cases were reported in the AS Phase 2/3 safety cohorts, PsA Phase 3 safety cohort (Table 3), or AS real-world cohort (Supplementary Table 10 in Additional File 4); tuberculosis IR was < 0.1 per 100 PY for the RA Phase 2/3 safety cohort (Table 3).

#### Herpes zoster

Herpes zoster (HZ) occurred in five (1.6%) and seven (1.7%) patients in the 48-week only-tofacitinib 5 mg BID and all-tofacitinib cohorts, respectively (Table 3); all cases involved a single dermatome, except for one patient (tofacitinib 10 mg BID) with HZ involving two adjacent dermatomes (this did not meet opportunistic infection criteria on adjudication). All cases of HZ were non-serious.

HZ IRs (95% CIs) were 2.18 (0.71–5.08) and 2.68 (1.08–5.53) per 100 PY for the 48-week only-tofacitinib 5 mg BID and all-tofacitinib cohorts, respectively (Table 3). These were similar vs. the RA Phase 2/3 and PsA Phase 3 safety cohorts (Table 3), and numerically higher vs. the AS real-world cohort (overlapping 95% CIs [Supplementary Table 10 in Additional File 4]).

#### Malignancies

No malignancies (excluding nonmelanoma skin cancer [NMSC]) or NMSCs were reported in the AS Phase 2/3 safety cohorts (Table 3). Across the RA Phase 2/3 and PsA Phase 3 safety cohorts, and AS real-world cohort, malignancies (excluding NMSC) and NMSCs occurred in 0–0.9% and 0–0.4% of patients, respectively; and IRs of malignancies (excluding NMSC) and NMSCs were all < 1.5 and < 0.5 per 100 PY, respectively, with 95% CIs overlapping (Table 3; Supplementary Table 10 in Additional File 4).

#### Cardiovascular events

No MACE, deep vein thrombosis (DVT), pulmonary embolism (PE), VTE, or arterial thromboembolism events (ATE) were reported in the AS Phase 2/3 safety cohorts (Table 3). Among the RA Phase 2/3 and PsA Phase 3 safety cohorts, and AS real-world cohort, MACE, VTE, DVT, PE, and ATE occurred in 0.2–0.4%, 0–0.3%, 0–0.2%, 0–0.1%, and 0–0.3% of patients, respectively, and IRs were all generally < 1 per 100 PY, with 95% CIs overlapping (Table 3; Supplementary Table 10 in Additional File 4).

#### Gastrointestinal perforations

No gastrointestinal perforations were observed in the AS Phase 2/3 safety cohorts, RA Phase 2/3 safety cohort, or AS real-world safety cohort (Table 3; Supplementary Table 10 in Additional File 4); gastrointestinal perforation IR (95% CI) was 0.50 (0.01–2.77) per 100 PY for the PsA Phase 2/3 safety cohort (Table 3).

#### Liver parameters, hepatic-related AEs, and drug-induced liver injury (AS Phase 2/3 safety cohorts only)

**Fig. 3:**
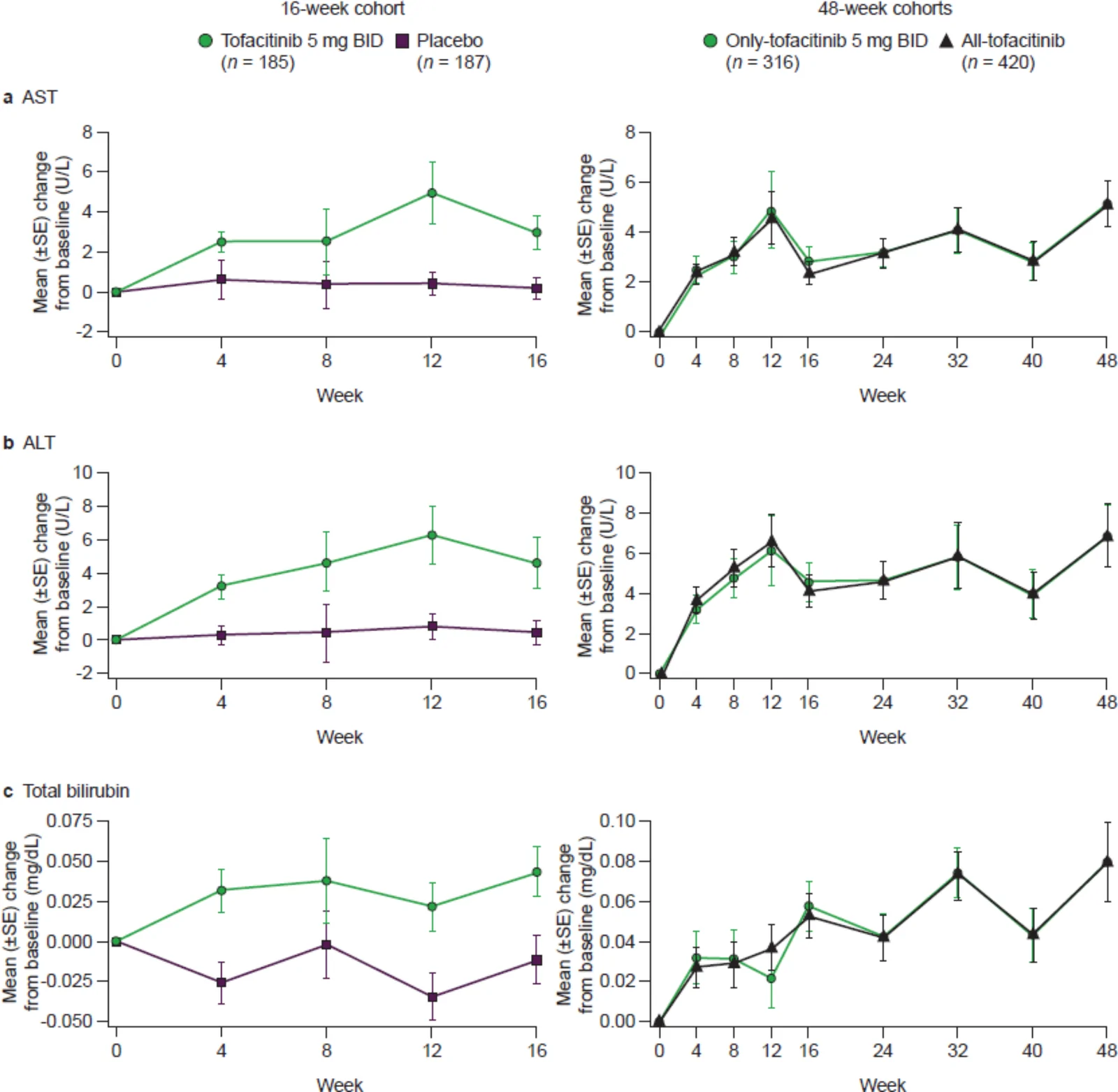
Change from baseline in AST, ALT, and total bilirubin with tofacitinib in AS Phase 2/3 safety cohorts. Baseline was defined as the last non-missing assessment prior to the first dose of investigational product (including placebo) in the 16-week placebo-controlled cohort and prior to the first dose of tofacitinib in the 48-week cohorts. Within the 16-week placebo-controlled and 48-week cohorts, patients in the Phase 2 RCT received tofacitinib to week 12. *ALT* alanine aminotransferase, *AS* ankylosing spondylitis, *AST* aspartate aminotransferase, *BID* twice daily, *n* total number of patients, *SE* standard error

In the tofacitinib 5 mg BID arm of the 16-week placebo-controlled cohort, aspartate aminotransferase and alanine aminotransferase levels increased from baseline to week 12, then decreased to week 16, while total bilirubin levels generally increased from baseline to week 16; overall, similar trends to week 16 were seen in the 48-week cohorts, with levels stabilizing from weeks 16 to 40, then increasing to week 48 (Fig. 3). Absolute values for liver parameters over time are shown in Supplementary Fig. 1 in Additional File 5 and patients with laboratory values meeting monitoring/discontinuation criteria are summarized in Supplementary Table 11 in Additional File 5. There were no events of liver enzyme elevations that were adjudicated as probable drug-induced liver injuries.

Supplementary Table 12 in Additional File 5 summarizes patients with confirmed transaminase and bilirubin elevations. There were no Hy’s Law cases.

### Change from baseline in laboratory values and vital signs in the AS Phase 2/3 safety cohorts

#### Hematologic parameters

Absolute values and changes from baseline for hematology parameters over time are shown in Supplementary Figs. 2 and 3 (in Additional File 6), respectively. For all AS Phase 2/3 safety cohorts, there were no study drug discontinuations due to hemoglobin reductions, neutropenia, lymphopenia, or thrombocytopenia (Supplementary Table 11 in Additional File 5).

#### Lipid parameters

Absolute values for total cholesterol, high-density and low-density lipoprotein cholesterol, and triglycerides over time are shown in Supplementary Fig. 4 in Additional File 6. In the tofacitinib 5 mg BID arm of the 16-week placebo-controlled cohort, levels increased to week 4, then were generally sustained to week 16; overall, similar trends were observed for the first 16 weeks of the 48-week tofacitinib cohorts, with levels sustained to week 48.

#### Creatine kinase and serum creatinine

Absolute values and changes from baseline for creatine kinase and serum creatinine over time are shown in Supplementary Figs. 5 and 6 (in Additional File 6), respectively. No patients met drug discontinuation criteria for creatine kinase or serum creatinine (Supplementary Table 11 in Additional File 5).

#### Vital signs

There were no clinically meaningful changes over time in heart rate (data not shown), electrocardiogram parameters (data not shown), or blood pressure (Supplementary Fig. 7 in Additional File 6).

Hypertension was reported by four (2.2%) and two (1.1%) patients receiving tofacitinib 5 mg BID and placebo, respectively, in the 16-week placebo-controlled cohort, and by nine (2.9%) and 11 (2.6%) patients in the 48-week only-tofacitinib 5 mg BID and all-tofacitinib cohorts, respectively (all hypertension AEs were mild/moderate).

Changes from baseline in body weight over time are shown in Supplementary Fig. 8 in Additional File 6; in the 16-week placebo-controlled cohort, greater increases in body weight were observed for tofacitinib 5 mg BID vs. placebo; generally, in the 48-week cohorts, body weight increased to week 16, then stabilized thereafter to week 48.

## Discussion

In this integrated analysis of Phase 2/3 RCTs [8, 9] of patients with AS receiving tofacitinib up to 48 weeks, no deaths, MACE, thromboembolic events, malignancies, opportunistic infections, or gastrointestinal perforations occurred; one serious infection occurred, and < 2% of patients had HZ. Within the limitations of the differing studies, patient demographics, characteristics, and statistical precision resulting from the sample size of the tofacitinib AS program, these safety findings were generally consistent with the established safety profile of tofacitinib in the RA and PsA clinical programs, and with real-world data from a cohort of US patients with AS.

Given differences in patient populations and characteristics, serious AE IRs in the AS Phase 2/3 safety cohorts were lower vs. the RA Phase 2/3 and PsA Phase 3 safety cohorts analyzed here, and vs. tofacitinib-treated patients with RA, PsA, ulcerative colitis, and psoriasis in previous studies [20]. In our study, differences in demographics and concomitant medications between cohorts should be noted (e.g., the AS Phase 2/3 safety cohort included younger patients and a lower proportion of concomitant oral corticosteroid users vs. the RA Phase 2/3 and PsA Phase 3 safety cohorts).

No cases of malignancies or MACE occurred in the AS Phase 2/3 safety cohorts (noting the short analysis period [up to 48 weeks]). Considering the overlapping 95% CIs, IRs of malignancies/MACE were generally similar between AS Phase 2/3 safety cohorts, RA Phase 2/3 and PsA Phase 3 safety cohorts, and the AS real-world cohort; this complements a previous report that IRs of malignancies/MACE are similar for tofacitinib-treated patients with RA, PsA, psoriasis, and ulcerative colitis [20].

The post-authorization safety RCT ORAL Surveillance reported an increased rate of MACE and malignancies (excluding NMSC) with tofacitinib vs. tumor necrosis factor inhibitors (TNFi) in patients with RA aged ≥ 50 years and with ≥ 1 CV risk factor [15]. While patients with AS have increased CV risk vs. the general population [5–7], risk for certain CV events may be lower in patients with AS vs. RA [21, 22]. Additionally, although it is generally accepted that patients with RA have an increased risk of certain malignancies, including lung cancer and lymphoma [23, 24], associations between AS and malignancies are less clear [25]. A recent cross-sectional study reported no significant differences in any-type-cancer risk between patients with AS and controls without rheumatic disease [26]. Differences in epidemiological and CV risk profiles between patients with AS and RA should be considered; e.g., AS populations are generally younger and include more males. In ORAL Surveillance, the increased risk of MACE/malignancies with tofacitinib vs. TNFi was more prominent in patients with RA aged ≥ 65 vs. < 65 years old [15]. Recent post hoc analyses of ORAL Surveillance identified risk factors that account for excessive risk of safety outcomes with tofacitinib vs. TNFi (i.e., differentiating risk factors). Kristensen et al. found that an increased risk of MACE, malignancies (excluding NMSC), and VTE with tofacitinib vs. TNFi was confined to patients who were either aged ≥ 65 years old or were current or past long-time smokers, and did not detect a difference in risk in patients aged < 65 years old who had never smoked [17]. Charles-Schoeman et al. reported that 15% of patients in ORAL Surveillance had HxASCVD, and in these, MACE risk was greater with tofacitinib vs. TNFi, per the primary study analysis; however, MACE risk appeared similar between tofacitinib and TNFi in patients without HxASCVD [18]. In ORAL Surveillance and consistently across the tofacitinib RA, PsA, and ulcerative colitis development programs, rates of these safety outcomes were low in patients without these risk factors (i.e., patients < 65 years old and never smokers, and patients with no HxASCVD [specifically for MACE]) [16, 17]. In the analysis of AS Phase 2/3 cohorts, < 5% of patients had HxASCVD and 3.1% were ≥ 65 years old, and 48.3% were current/past smokers, however the duration of exposure was short. Accordingly, there is currently a paucity of long-term data on the occurrence of major and rare safety outcomes with tofacitinib in patients with AS who have differentiating risk factors. As there are no prospective studies of sufficient size and duration and with risk-enrichment as in ORAL Surveillance to compare the safety of tofacitinib or other JAK inhibitors with TNFi in patients with AS [16], it is appropriate to apply the results from ORAL Surveillance to patients with AS, particularly those with differentiating risk factors for MACE, malignancy, and VTE (i.e., older age [≥ 65 years old], current or past long-time smokers, or, specifically for MACE, HxASCVD) [17, 18, 27]. As recently described in a review of current clinical data on use of tofacitinib in PsA and AS and in line with the current labeling for JAK inhibitors, these readily identifiable risk factors can form a framework for an individualized risk factor-based approach to clinical decision-making on treatment with tofacitinib or other JAK inhibitor vs. TNFi [16].

Serious infection IRs were numerically lower in the AS 48-week only-tofacitinib 5 mg BID and all-tofacitinib cohorts vs. the RA Phase 2/3 and PsA Phase 3 safety cohorts analyzed here, and vs. tofacitinib-treated patients with RA, PsA, ulcerative colitis, and psoriasis in other studies [20]; this could reflect differences in demographics or concomitant medications across cohorts/diseases. Serious infection IRs were greater in the AS real-world cohort of bDMARD initiators vs. the tofacitinib AS Phase 2/3 cohorts, and were similar to those reported in patients with AS newly treated with TNFi in a claims-based cohort study [28]. Serious infection IRs have been shown to be higher with tofacitinib vs. TNFi in CV-risk-enriched patients with RA (ORAL Surveillance) [15, 29].

Use of JAK inhibitors is associated with an increased risk of HZ (serious and non-serious) in patients with immune-mediated inflammatory diseases [30–33]. In our analysis of patients with AS, there were no HZ cases in the 16-week placebo-controlled cohort, and HZ IRs in the 48-week cohorts were numerically similar vs. the RA Phase 2/3 and PsA Phase 3 safety cohorts. However, HZ IRs with tofacitinib were greater vs. the AS real-world cohort of bDMARD initiators; it is possible that HZ is under-reported in real-world populations. Nevertheless, our data confirm that the HZ risk associated with tofacitinib applies to patients with AS; and our findings are consistent with the higher incidence of HZ with tofacitinib vs. TNFi observed in patients with RA in ORAL Surveillance [15, 29].

Patients with AS often experience extramusculoskeletal manifestations, including uveitis, inflammatory bowel disease, and psoriasis [34]. For all AS Phase 2/3 safety cohorts analyzed here, uveitis occurred in < 2% of tofacitinib-treated patients, similar to the frequency with placebo (16-week placebo-controlled cohort), and consistent with the frequency of uveitis reported in secukinumab-treated patients with AS [35].

The main limitations of this analysis of AS Phase 2/3 RCTs include the small patient numbers, the small number of events for some safety outcomes, the lack of long-term extension data, and the limited extent and duration of exposure (e.g., only 233 PY of exposure for the 48-week all-tofacitinib cohort). Furthermore, comparisons between tofacitinib and placebo could only be made to week 16. There is a high degree of statistical uncertainty associated with the presented IRs (i.e., wide and overlapping 95% CIs) rendering it difficult to compare safety outcomes across treatments/cohorts. Care must be taken when comparing safety outcomes in patients with AS vs. RA/PsA, due to differences in demographics/disease characteristics. Similarly, differences in AE reporting between RCTs and real-world databases should be noted. For example, the AS real-world cohort was based on the US MarketScan database, in which AEs are recorded using administrative codes; therefore, some outcomes may be misclassified. Furthermore, RCTs have strict inclusion and exclusion criteria, whereas real-world databases comprise more diverse populations; however, in the current analyses, similar exclusion criteria to the AS Phase 3 RCT were applied to the AS real-world cohort, to mitigate this limitation. Of note, the AS real-world cohort included US patients while the AS Phase 2/3 safety cohorts included patients from multiple geographical locations. Most patients in the AS Phase 2/3 safety cohorts were not at high risk for TEAEs, such as ASCVD events, and it is possible that patients at higher risk may have experienced a different profile of TEAEs. Other limitations are that dose-response relationships could not be formally tested, and propensity scoring, to avoid confounding bias, was not performed.

## Conclusions

The results from this integrated safety analysis of data from the tofacitinib AS Phase 2/3 RCTs demonstrated that tofacitinib 5 mg BID is well tolerated to 48 weeks in patients with active AS. Overall, the safety profile of tofacitinib in this analysis was consistent with the established safety profile observed in patients with RA and PsA treated with tofacitinib, and in real-world patients with AS initiating bDMARDs in the US.

## Electronic supplementary material

Below is the link to the electronic supplementary material.

Supplementary Material 1

## Data Availability

Upon request, and subject to review, Pfizer will provide the data that support the findings of this study. Subject to certain criteria, conditions, and exceptions, Pfizer may also provide access to the related individual de-identified participant data. See https://www.pfizer.com/science/clinical-trials/trial-data-and-results for more information.

## Abbreviations

AE: Adverse event
AS: Ankylosing spondylitis
ASCVD: Atherosclerotic cardiovascular disease
ATE: Arterial thromboembolism event
bDMARD: Biologic disease-modifying antirheumatic drug
BID: Twice daily
CI: Confidence interval
csDMARD: Conventional synthetic disease-modifying antirheumatic drug
CV: Cardiovascular
DMARD: Disease-modifying antirheumatic drug
DVT: Deep vein thrombosis
HZ: Herpes zoster
Hx: History
IR: Incidence rate
MACE: Major adverse cardiovascular event
NMSC: Nonmelanoma skin cancer
NSAID: Nonsteroidal anti-inflammatory drug
PE: Pulmonary embolism
PsA: Psoriatic arthritis
PY: Patient-year
RA: Rheumatoid arthritis
RCT: Randomized controlled trial
TEAE: Treatment-emergent adverse events
TNFi: Tumor necrosis factor inhibitor
VTE: Venous thromboembolism
