# Supplementary material for: Integrated safety analysis of tofacitinib from Phase 2 and 3 trials of patients with ankylosing spondylitis
Source: Adv Rheumatol. 2024 Dec 18;64:87. doi: 10.1186/s42358-024-00402-x (PMC13488738; doi:10.1186/s42358-024-00402-x)
Supplement: Supplementary file 1 [file 42358_2024_402_MOESM1_ESM.pdf]

Supplementary Table 1. RCTs included in the RA Phase 2/3 and PsA Phase 3 safety cohorts defined in the current post hoc analysis.

Clinicaltrials.gov identifier [Ref]	Protocol number	Patient population	Tofacitinib doses	Control arm	Study duration
RA Phase 2/3 safety cohort					
Phase 2					
NCT00147498 [2]	A3921019	Active RA with inadequate or unacceptable toxicity to either MTX 15 mg or greater, or to any of the following: etanercept, infliximab, or adalimumab	5 mg BID, 15 mg BID, or 30 mg BID monotherapy	Placebo BID	6 weeks of treatment; 6 weeks of follow up
NCT00413660 [3]	A3921025	Active RA with inadequate response to MTX	1, 3, 5, 10, or 15 mg BID or 20 mg QD with background MTX	Placebo	24 weeks
NCT00550446 [4]	A3921035	Active RA with inadequate response to ≥ 1 DMARD	1, 3, 5, 10, or 15 mg BID monotherapy	Adalimumab SC 40 mg Q2W; placebo	24 weeks
NCT00603512 [5]	A3921039	Active RA with inadequate response to MTX	1, 3, 5, or 10 mg BID plus background MTX	Placebo	12 weeks
NCT00687193 [6]	A3921040	Active RA with inadequate response to ≥ 1 DMARD	1, 3, 5, 10, or 15 mg BID monotherapy	Placebo	12 weeks
NCT01164579 [7]	A3921068	Early active RA, MTX-naïve	10 mg BID plus MTX or 10 mg BID monotherapy	MTX	12 months
NCT00976599 [8]	A3921073	Active RA with inadequate response to MTX	10 mg BID plus background MTX	Placebo	4 weeks

NCT01359150 [9]	A3921129	Active RA	10 mg BID monotherapy (half of patients) or with background MTX; vaccinated (pneumococcal and influenza) 4 weeks later	Placebo only (half of patients) or placebo plus MTX	9 weeks
NCT02147587 [10]	A3921237	Moderate to severe RA with inadequate response to MTX	5 mg BID plus background MTX; 2–3 weeks post-herpes zoster vaccination	Placebo	14 weeks
Phase 3					
NCT00960440 [11]	ORAL Step, A3921032	Moderate to severe RA with inadequate response to TNFi	5 or 10 mg BID with background MTX	Placebo (advanced to tofacitinib at month 3)	6 months
NCT00847613 [12]	ORAL Scan, A3921044	Active RA with inadequate response to MTX	5 or 10 mg BID with background MTX	Placebo (advanced to tofacitinib at month 3 [nonresponders] or month 6 [remaining patients])	24 months
NCT00814307 [13]	ORAL Solo, A3921045	Active RA with inadequate response to ≥ 1 DMARD	5 or 10 mg BID monotherapy	Placebo (advanced to tofacitinib at month 3)	6 months
NCT00856544 [14]	ORAL Sync, A3921046	Active RA with inadequate response to ≥ 1 DMARD	5 or 10 mg BID with background nonbiologic DMARD	Placebo (advanced to tofacitinib at month 3 [nonresponders] or month 6 [remaining patients])	12 months
NCT00853385 [15]	ORAL Standard, A3921064	Active RA with incomplete response to MTX	5 or 10 mg BID with background MTX	Adalimumab 40 mg SC Q2W; placebo (patients receiving placebo were advanced to tofacitinib at month 3 [nonresponders] or month 6 [remaining patients])	12 months

NCT01039688 [16]	ORAL Start, A3921069	Active RA, MTX-naïve	5 or 10 mg BID monotherapy	MTX	24 months
NCT02187055 [17]	ORAL Strategy, A3921187	Moderately to severely active RA with inadequate response to MTX	5 mg BID monotherapy or with background MTX	Adalimumab 40 mg SC Q2W with background MTX	12 months
PsA Phase 3 safety cohorts					
Phase 3					
NCT01882439 [18]	OPAL Beyond, A3921125	Active PsA with inadequate response to ≥ 1 TNFi	5 or 10 mg BID, with background csDMARD	Placebo BID (advanced to tofacitinib at month 3) with background csDMARD	6 months
NCT01877668 [19]	OPAL Broaden, A3921091	Active PsA with inadequate response to ≥ 1 csDMARD	5 or 10 mg BID, with background csDMARD	Adalimumab 40 mg SC Q2W with background csDMARD; placebo BID (advanced to tofacitinib at month 3) with background csDMARD	12 months

BID, twice daily; csDMARD, conventional synthetic disease-modifying antirheumatic drug; DMARD, disease-modifying antirheumatic drug; MTX, methotrexate; PsA, psoriatic arthritis; Q2W, once every 2 weeks; QD, once daily; RA, rheumatoid arthritis; RCT, randomized controlled trial; SC, subcutaneous; TNFi, tumor necrosis factor inhibitors

References

1. Deodhar A, Sliwinska-Stanczyk P, Xu H, Baraliakos X, Gensler LS, Fleishaker D, Wang L, Wu J, Menon S, Wang C, Dina O, Fallon L, Kanik KS, van der Heijde D (2021) Tofacitinib for the treatment of ankylosing spondylitis: a phase III, randomised, double-blind, placebo-controlled study. Ann Rheum Dis 80:1004–1013. 10.1136/annrheumdis-2020-219601
2. Kremer JM, Bloom BJ, Breedveld FC, Coombs JH, Fletcher MP, Gruben D, Krishnaswami S, Burgos-Vargas R, Wilkinson B, Zerbini CAF, Zwillich SH (2009) The safety and efficacy of a JAK inhibitor in patients with active rheumatoid arthritis: results of a double-blind, placebo-controlled phase IIa trial of three dosage levels of CP-690,550 versus placebo. Arthritis Rheum 60:1895–1905. 10.1002/art.24567
3. Kremer JM, Cohen S, Wilkinson BE, Connell CA, French JL, Gomez-Reino J, Gruben D, Kanik KS, Krishnaswami S, Pascual-Ramos V, Wallenstein G, Zwillich SH (2012) A phase IIb dose-ranging study of the oral JAK inhibitor tofacitinib (CP-690,550) versus placebo in combination with background methotrexate in patients with active rheumatoid arthritis and an inadequate response to methotrexate alone. Arthritis Rheum 64:970–981. 10.1002/art.33419
4. Fleischmann R, Cutolo M, Genovese MC, Lee EB, Kanik KS, Sadis S, Connell CA, Gruben D, Krishnaswami S, Wallenstein G, Wilkinson BE, Zwillich SH (2012) Phase IIb dose-ranging study of the oral JAK inhibitor tofacitinib (CP-690,550) or adalimumab monotherapy versus placebo in patients with active rheumatoid arthritis with an inadequate response to disease-modifying antirheumatic drugs. Arthritis Rheum 64:617–629. 10.1002/art.33383
5. Tanaka Y, Takeuchi T, Yamanaka H, Suzuki M, Nakamura H, Toyoizumi S, Bradley J, Zwillich S (2011) Tofacitinib (CP-690,550), an oral Janus kinase inhibitor, as

monotherapy in Japanese patients with active rheumatoid arthritis: a 12-week phase 2b study. Arthritis Rheum Accessed

6. Tanaka Y, Takeuchi T, Yamanaka H, Nakamura H, Toyoizumi S, Zwillich S (2015) Efficacy and safety of tofacitinib as monotherapy in Japanese patients with active rheumatoid arthritis: a 12-week, randomized, phase 2 study. Mod Rheumatol 25:514–521. 10.3109/14397595.2014.995875
7. Conaghan PG, Østergaard M, Bowes MA, Wu C, Fuerst T, van der Heijde D, Irazoque-Palazuelos F, Soto-Raices O, Hrycaj P, Xie Z, Zhang R, Wyman BT, Bradley JD, Soma K, Wilkinson B (2016) Comparing the effects of tofacitinib, methotrexate and the combination, on bone marrow oedema, synovitis and bone erosion in methotrexate-naive, early active rheumatoid arthritis: results of an exploratory randomised MRI study incorporating semiquantitative and quantitative techniques. Ann Rheum Dis 75:1024–1033. 10.1136/annrheumdis-2015-208267
8. Boyle DL, Soma K, Hodge J, Kavanaugh A, Mandel D, Mease P, Shurmur R, Singhal AK, Wei N, Rosengren S, Kaplan I, Krishnaswami S, Luo Z, Bradley J, Firestein GS (2015) The JAK inhibitor tofacitinib suppresses synovial JAK1-STAT signalling in rheumatoid arthritis. Ann Rheum Dis 74:1311–1316. 10.1136/annrheumdis-2014-206028
9. Winthrop KL, Silverfield J, Racewicz A, Neal J, Lee EB, Hrycaj P, Gomez-Reino J, Soma K, Mebus C, Wilkinson B, Hodge J, Fan H, Wang T, Bingham 3rd CO (2016) The effect of tofacitinib on pneumococcal and influenza vaccine responses in rheumatoid arthritis. Ann Rheum Dis 75:687–695. 10.1136/annrheumdis-2014-207191
10. Winthrop KL, Wouters AG, Choy EH, Soma K, Hodge JA, Nduaka CI, Biswas P, Needle E, Passador S, Mojcik CF, Rigby WF (2017) The safety and immunogenicity

of live zoster vaccination in patients with rheumatoid arthritis before starting tofacitinib: a randomized phase II trial. Arthritis Rheumatol 69:1969–1977. doi: 10.1002/art.40187

11. Burmester G, Charles-Schoeman C, Isaacs J, Hendrikx T, Kwok K, Zwillich S, Riese R (2013) Tofacitinib, an oral Janus kinase inhibitor: safety comparison in patients with rheumatoid arthritis and an inadequate response to nonbiologic or biologic disease modifying anti-rheumatic drugs. Ann Rheum Dis https://ard.bmj.com/content/72/Suppl_3/A245.2. Accessed
12. van der Heijde D, Tanaka Y, Fleischmann R, Keystone E, Kremer J, Zerbini C, Cardiel MH, Cohen S, Nash P, Song YW, Tegzová D, Wyman BT, Gruben D, Benda B, Wallenstein G, Krishnaswami S, Zwillich SH, Bradley JD, Connell CA, ORAL Scan Investigators (2013) Tofacitinib (CP-690,550) in patients with rheumatoid arthritis receiving methotrexate: twelve-month data from a twenty-four-month phase III randomized radiographic study. Arthritis Rheum 65:559–570. 10.1002/art.37816
13. Fleischmann R, Kremer J, Cush J, Schulze-Koops H, Connell CA, Bradley JD, Gruben D, Wallenstein GV, Zwillich SH, Kanik KS, ORAL Solo Investigators (2012) Placebo-controlled trial of tofacitinib monotherapy in rheumatoid arthritis. N Engl J Med 367:495–507. 10.1056/NEJMoa1109071
14. Kremer J, Li Z-G, Hall S, Fleischmann R, Genovese M, Martin-Mola E, Isaacs JD, Gruben D, Wallenstein G, Krishnaswami S, Zwillich SH, Koncz T, Riese R, Bradley J (2013) Tofacitinib in combination with nonbiologic disease-modifying antirheumatic drugs in patients with active rheumatoid arthritis: a randomized trial. Ann Intern Med 159:253–261. 10.7326/0003-4819-159-4-201308200-00006
15. van Vollenhoven RF, Fleischmann R, Cohen S, Lee EB, García Meijide JA, Wagner S, Forejtova S, Zwillich SH, Gruben D, Koncz T, Wallenstein GV, Krishnaswami S,

Bradley JD, Wilkinson B, for the ORAL Standard Investigators (2012) Tofacitinib or adalimumab versus placebo in rheumatoid arthritis. N Engl J Med 367:508–519. 10.1056/NEJMoa1112072

16. Lee EB, Fleischmann R, Hall S, Wilkinson B, Bradley J, Gruben D, Koncz T, Krishnaswami S, Wallenstein GV, Zang C, Zwillich SH, van Vollenhoven RF, for the ORAL Start investigators (2014) Tofacitinib versus methotrexate in rheumatoid arthritis. N Engl J Med 370:2377–2386. 10.1056/NEJMoa1310476
17. Fleischmann R, Mysler E, Hall S, Kivitz AJ, Moots RJ, Luo Z, DeMasi R, Soma K, Zhang R, Takiya L, Tatulych S, Mojcik C, Krishnaswami S, Menon S, Smolen JS, on behalf of the ORAL Strategy investigators (2017) Efficacy and safety of tofacitinib monotherapy, tofacitinib with methotrexate, and adalimumab with methotrexate in patients with rheumatoid arthritis (ORAL Strategy): a phase 3b/4, double-blind, head-to-head, randomised controlled trial. Lancet 390:457–468. 10.1016/S0140-6736(17)31618-5
18. Gladman D, Rigby W, Azevedo VF, Behrens F, Blanco R, Kaszuba A, Kudlacz E, Wang C, Menon S, Hendrikx T, Kanik KS (2017) Tofacitinib for psoriatic arthritis in patients with an inadequate response to TNF inhibitors. N Engl J Med 377:1525-1536. 10.1056/NEJMoa1615977
19. Mease P, Hall S, FitzGerald O, van der Heijde D, Merola JF, Avila-Zapata F, Ciéslak D, Graham D, Wang C, Menon S, Hendrikx T, Kanik KS (2017) Tofacitinib or adalimumab versus placebo for psoriatic arthritis. N Engl J Med 377:1537-1550. 10.1056/NEJMoa1615975

Supplementary Table 2. Cardiovascular risk factors at baseline for patients in the 48-week only-tofacitinib 5 mg BID and all-tofacitinib cohorts[a] (AS Phase 2/3 safety cohorts) with HxASCVD[b]; and in those without HxASCVD categorized by baseline 10-year ASCVD risk[c]

	Overall		HxASCVD		No HxASCVD							
					High (≥ 20%)[d]		Intermediate (≥ 7.5 – < 20%)[d]		Borderline (≥ 5 – < 7.5%)[d]		Low (< 5%)[d]	
	48-week only-tofacitinib 5 mg BID	48-week all-tofacitinib	48-week only-tofacitinib 5 mg BID	48-week all-tofacitinib	48-week only-tofacitinib 5 mg BID	48-week all-tofacitinib	48-week only-tofacitinib 5 mg BID	48-week all-tofacitinib	48-week only-tofacitinib 5 mg BID	48-week all-tofacitinib	48-week only-tofacitinib 5 mg BID	48-week all-tofacitinib
N (%, based on overall *N*)	316	420	11 (3.5)	15 (3.6)	5 (1.6)	7 (1.7)	43 (13.7)	51 (12.2)	17 (5.4)	29 (6.9)	239 (75.9)	316 (75.6)
Age ≥ 60 years, *n* (%)	25 (7.9)	36 (8.6)	5 (45.5)	6 (40.0)	1 (20.0)	3 (42.9)	17 (39.5)	22 (43.1)	2 (11.8)	4 (13.8)	0	1 (0.3)
Age ≥ 50 years, *n* (%)	75 (23.7)	102 (24.3)	8 (72.7)	11 (73.3)	5 (100.0)	7 (100.0)	35 (81.4)	42 (82.4)	9 (52.9)	16 (55.2)	17 (7.1)	25 (7.9)
Male, *n* (%)	261 (82.6)	333 (79.3)	9 (81.8)	11 (73.3)	4 (80.0)	6 (85.7)	37 (86.0)	43 (84.3)	13 (76.5)	22 (75.9)	197 (82.4)	249 (78.8)
BMI ≥ 30 kg/m^2, *n* (%)	71 (22.5)	95 (22.6)	2 (18.2)	4 (26.7)	1 (20.0)	2 (28.6)	16 (37.2)	20 (39.2)	7 (41.2)	10 (34.5)	45 (18.8)	59 (18.7)
Current/ past smoker, *n* (%)	100 (31.6)/ 51 (16.1)	136 (32.4)/ 67 (16.0)	1 (9.1)/ 3 (27.3)	3 (20.0)/ 3 (20.0)	3 (60.0)/ 0	4 (57.1)/ 0	22 (51.2)/ 10 (23.3)	26 (51.0)/ 12 (23.5)	9 (52.9)/ 2 (11.8)	16 (55.2)/ 4 (13.8)	65 (27.2)/ 36 (15.1)	87 (27.5)/ 48 (15.2)
History of diabetes mellitus, *n* (%)	12 (3.8)	18 (4.3)	1 (9.1)	1 (6.7)	3 (60.0)	3 (42.9)	5 (11.6)	8 (15.7)	1 (5.9)	3 (10.3)	2 (0.8)	3 (0.9)
History of hypertension, *n* (%)	78 (24.7)	96 (22.9)	6 (54.5)	9 (60.0)	4 (80.0)	5 (71.4)	26 (60.5)	31 (60.8)	8 (47.1)	12 (41.4)	34 (14.2)	39 (12.3)

History of hyperlipidemia, *n* (%)	26 (8.2)	34 (8.1)	4 (36.4)	5 (33.3)	3 (60.0)	3 (42.9)	8 (18.6)	9 (17.6)	0	1 (3.4)	11 (4.6)	16 (5.1)
History of VTE, *n* (%)	7 (2.2)	8 (1.9)	0	0	0	0	2 (4.7)	2 (3.9)	1 (5.9)	1 (3.4)	4 (1.7)	5 (1.6)
Antiplatelet use, *n* (%)[e]	11 (3.5)	16 (3.8)	5 (45.5)	7 (46.7)	1 (20.0)	1 (14.3)	2 (4.7)	4 (7.8)	1 (5.9)	2 (6.9)	2 (0.8)	2 (0.6)
Statin use, *n* (%)[e]	18 (5.7)	23 (5.5)	4 (36.4)	5 (33.3)	1 (20.0)	1 (14.3)	5 (11.6)	5 (9.8)	0	0	8 (3.3)	12 (3.8)

[a]Within the 48-week cohorts, patients in the Phase 2 RCT received tofacitinib to week 12
[b]HxASCVD was defined as ≥ 1 occurrence of CAD, CeVD, or PAD. History of CAD, CeVD, or PAD was identified in patients' general medical history through MedDRA Preferred Terms consistent with one of these conditions and reflecting prior/ongoing events, procedures, or diagnoses
[c]Baseline 10-year ASCVD risk was determined using the ASCVD-PCE risk calculator, developed by the American College of Cardiology/American Heart Association [1]. Scores were calculated based on patients' age, sex, race (White, Black, other), smoking status (yes/no), systolic blood pressure, antihypertensive treatment (yes/no), total cholesterol, high-density lipoprotein cholesterol, and diabetes (yes/no)
[d]Percentages were based on all patients with non-missing values. Patients without HxASCVD but with missing ASCVD-PCE risk data: 48-week only-tofacitinib 5 mg BID cohort, *n* = 1; 48-week all-tofacitinib cohort, *n* = 2
[e]Day 1 use
AS, ankylosing spondylitis; ASCVD, atherosclerotic cardiovascular disease; BID, twice daily; BMI, body mass index; CAD, coronary artery disease; CeVD, cerebrovascular disease; Hx, history (of); MedDRA, Medical Dictionary for Regulatory Activities; *N*, total number of patients included in analysis; *n*, number of patients with each characteristic; PAD, peripheral artery disease; PCE, pooled cohort equations; RCT, randomized controlled trial; VTE, venous thromboembolism

Reference:

1. Arnett DK, Blumenthal RS, Albert MA, Buroker AB, Goldberger ZD, Hahn EJ, Himmelfarb CD, Khera A, Lloyd-Jones D, McEvoy JW et al. 2019 ACC/AHA Guideline on the Primary Prevention of Cardiovascular Disease: A Report of the American College of Cardiology/American Heart Association Task Force on Clinical Practice Guidelines. Circulation. 2019;140(11):e596-e646.

Supplementary Table 3 Demographics and baseline disease characteristics of the AS real-world cohort[a]

	N = 2253
Age (years), mean (SD)	43.1 (12.0)
≥ 65 years, *n* (%)	51 (2.3)
Male, *n* (%)	1379 (61.2)
Region in US, *n* (%)	
North-Central	351 (15.6)
North-East	353 (15.7)
South	937 (41.6)
West	556 (24.7)
Missing	56 (2.5)
Medical history, *n* (%)	
Coronary heart disease	0 (0.0)
VTE (PE or DVT)	15 (0.7)
Hypertension (based on diagnosis codes)	565 (25.1)
Inflammatory bowel disease	0 (0.0)
Prior medication, *n* (%)	
bDMARD	891 (39.5)
TNFi	891 (39.5)
Adalimumab	362 (16.1)
Etanercept	412 (18.3)
csDMARD	677 (30.0)
Oral corticosteroids[b]	966 (42.9)
Concomitant medication, *n* (%)	
Non-COX NSAIDs	1547 (68.7)
COX NSAIDs	323 (14.3)

[a]Exclusion criteria similar to those within the tofacitinib AS Phase 3 RCT were applied. Patients were bDMARD initiators who were bDMARD-naïve or -experienced as of index date (date of first prescription or administration for AS treatment, or first procedure date after confirmation of AS diagnosis), with data censored

at 16 or 48 weeks for the first initiation
[b]Use determined as within 90 days of index date
AS, ankylosing spondylitis; bDMARD: biologic disease-modifying antirheumatic drug; COX, cyclooxygenase; csDMARD, conventional synthetic disease-modifying antirheumatic drug; DVT, deep vein thrombosis; *N*, total number of patients included in analysis; *n*, number of patients in each analysis category; NSAID, nonsteroidal anti-inflammatory drug; PE, pulmonary embolism; RCT, randomized controlled trial; SD, standard deviation; TNFi, tumor necrosis factor inhibitors; VTE, venous thromboembolism.

Supplementary Table 4. Summary of all TEAEs, serious AEs, severe AEs, and discontinuations due to AEs for patients in the 48-week only-tofacitinib 5 mg BID and all-tofacitinib cohorts[a] (AS Phase 2/3 safety cohorts), by age.

	48-week only-tofacitinib 5 mg BID cohort		48-week all-tofacitinib cohort	
	< 65 years (*N* = 309)	≥ 65 years (*N* = 7)	< 65 years (*N* = 407)	≥ 65 years (*N* = 13)
n (%)<br>*IR [95% CI, events per 100 PY]*				
TEAEs	193 (62.5)<br>157.39 [135.97–181.23]	5 (71.4)<br>145.98 [47.40–340.68]	241 (59.2)<br>169.01 [148.34–191.75]	7 (53.9)<br>143.88 [57.85–296.45]
Serious AEs	8 (2.6)<br>3.58 [1.54–7.05]	0<br>0.00 [0.00–65.18]	8 (2.0)<br>3.16 [1.36–6.22]	1 (7.7)<br>13.61 [0.34–75.82]
Severe AEs	7 (2.3)<br>3.13 [1.26–6.44]	0<br>0.00 [0.00–65.18]	8 (2.0)<br>3.16 [1.36–6.22]	0<br>[0.00–48.8]
Discontinuation due to AEs[b]	10 (3.2)<br>4.45 [2.13–8.18]	1 (14.3)<br>17.68 [0.45–98.50]	11 (2.7)<br>4.32 [2.16–7.74]	1 (7.7)<br>13.23 [0.34–73.73]

MedDRA version 23.0 coding dictionary applied. IRs (patients with events/100 PY), 95% CIs, and *n* (%) based on a 28-day risk period (time of first to last study drug dose +28 days). For patients who switched from placebo to tofacitinib 5 mg BID after week 16, the first dose date refers to the date of the first tofacitinib dose. IRs for the 48-week cohorts are estimated without adjusting for study. Exact Poisson (adjusted for PY) 95% CI are provided for IRs

[a]Within the 48-week cohorts, patients in the Phase 2 RCT received tofacitinib to week 12

[b]Discontinuation due to AEs is a mixture of discontinuation of study (Phase 2 RCT) and discontinuation of study treatment (Phase 3 RCT) due to AEs

AE, adverse event; AS, ankylosing spondylitis; BID, twice daily; CI, confidence interval; IR, incidence rate; MedDRA, Medical Dictionary for Regulatory Activities; *N*, total number of patients included in analysis; *n*, number of patients with event; PY, patient-years; RCT, randomized controlled trial; TEAE, treatment-emergent adverse event

Supplementary Table 5. Summary of all TEAEs, serious AEs, severe AEs, and discontinuations due to AEs for patients in the 48-week only-tofacitinib 5 mg BID and all-tofacitinib cohorts[a] (AS Phase 2/3 safety cohorts), by sex.

	48-week only-tofacitinib 5 mg BID cohort		48-week all-tofacitinib cohort	
	Male<br>(*N* = 261)	Female<br>(*N* = 55)	Male<br>(*N* = 333)	Female<br>(*N* = 87)
n (%)<br>*IR [95% CI, events per 100 PY]*				
All TEAEs	160 (61.3)<br>148.59 [126.46–173.48]	38 (69.1)<br>206.85 [146.38–283.92]	189 (56.8)<br>152.65 [131.66–176.03]	59 (67.8)<br>249.48 [189.91–321.81]
Serious AEs	6 (2.3)<br>3.10 [1.14–6.74)	2 (3.6)<br>5.62 [0.68–20.29]	7 (2.1)<br>3.25 [1.31–6.69]	2.(2.3)<br>4.43 [0.54–16.01]
Severe AEs	6 (2.3)<br>3.10 [1.14–6.76]	1 (1.8)<br>2.76 [0.07–15.93]	6 (1.8)<br>2.79 [10.2–6.07]	2 (2.3)<br>4.39 [0.53–15.85]
Discontinuation due to AEs[b]	8 (3.1)<br>4.11 [1.78–8.11]	3 (5.5)<br>8.31 [1.71–24.29]	9 (2.7)<br>4.16 [1.90–7.90]	3 (3.5)<br>6.58 [1.36–19.22]

MedDRA version 23.0 coding dictionary applied. IRs (patients with events/100 PY), 95% CIs, and *n* (%) based on a 28-day risk period (time of first to last study drug dose +28 days). For patients who switched from placebo to tofacitinib 5 mg BID after week 16, the first dose date refers to the date of the first tofacitinib dose. IRs for the 48-week cohorts are estimated without adjusting for study. Exact Poisson (adjusted for PY) 95% CI are provided for IRs

[a]Within the 48-week cohorts, patients in the Phase 2 RCT received tofacitinib to week 12

[b]Discontinuation due to AEs is a mixture of discontinuation of study (Phase 2 RCT) and discontinuation of study treatment (Phase 3 RCT) due to AEs

AE, adverse event; AS, ankylosing spondylitis; BID, twice daily; CI, confidence interval; IR, incidence rate; MedDRA, Medical Dictionary for Regulatory Activities; *N*, total number of patients included in analysis; *n*, number of patients with event; PY, patient-years; RCT, randomized controlled trial; TEAE, treatment-emergent adverse event

Supplementary Table 6. Summary of all TEAEs, serious AEs, severe AEs, and discontinuations due to AEs for patients in the 48-week only-tofacitinib 5 mg BID and all-tofacitinib cohorts[a] (AS Phase 2/3 safety cohorts), by race.

	48-week only-tofacitinib 5 mg BID cohort			48-week all-tofacitinib cohort		
	White (*N* = 252)	Asian (*N* = 63)	Other (*N* = 1)	White (*N* = 334)	Asian (*N* = 85)	Other (*N* = 1)
n (%) *IR [95% CI, events per 100 PY]*						
TEAE	149 (59.1) 136.90 [115.80–160.73]	49 (77.8) 302.22 [223.58–399.55]	0 0.00 [0.00–369.14]	183 (54.8) 144.33 [124.18–166.82]	65 (76.5) 330.47 [255.05–421.22]	0 0.00 [0.00–369.14]
Serious AEs	6 (2.4) 3.28 [1.20–7.15]	2 (3.2) 4.38 [0.53–15.83]	0 0.00 [0.00–369.14]	7 (2.1) 3.38 [1.36–6.97]	2 (2.4) 3.80 [0.46–13.73]	0 0.00 [0.00–369.14]
Severe AEs	7 (2.8) 3.84 [1.54–7.91]	0 0.00 [0.00–8.00]	0 0.00 [0.00–369.14]	8 (2.4) 3.87 [1.67–7.62]	0 0.00 [0.00–6.95]	0 0.00 [0.00–369.14]
Discontinuation due to AEs[b]	10 (4.0) 5.45 [2.61–10.02]	1 (1.6) 2.17 [0.06–12.11]	0 0.00 [0.00–369.14]	11 (3.3) 5.29 [2.64–9.46]	1 (1.2) 1.89 [0.05–10.51]	0 0.00 [0.00–369.14]

MedDRA version 23.0 coding dictionary applied. IRs (patients with events/100 PY), 95% CIs, and *n* (%) based on a 28-day risk period (time of first to last study drug dose +28 days). For patients who switched from placebo to tofacitinib 5 mg BID after week 16, the first dose date refers to the date of the first tofacitinib dose. IRs for the 48-week cohorts are estimated without adjusting for study. Exact Poisson (adjusted for PY) 95% CI are provided for IRs.

[a]Within the 48-week cohorts, patients in the Phase 2 RCT received tofacitinib to week 12.

[b]Discontinuation due to AEs is a mixture of discontinuation of study (Phase 2 RCT) and discontinuation of study treatment (Phase 3 RCT) due to AEs

AE, adverse event; AS, ankylosing spondylitis; BID, twice daily; CI, confidence interval; IR, incidence rate; MedDRA, Medical Dictionary for Regulatory Activities; *N*, total number of patients included in analysis; *n*, number of patients with event; PY, patient-years; RCT, randomized controlled trial; TEAE, treatment-emergent adverse event

Supplementary Table 7. Summary of all TEAEs, serious AEs, severe AEs, and discontinuations due to AEs in patients for patients in the 48-week only-tofacitinib 5 mg BID and all-tofacitinib cohorts[a] (AS Phase 2/3 safety cohorts), by geographical region.

	48-week only-tofacitinib 5 mg BID cohort				48-week all-tofacitinib cohort			
	North America (*N* = 38)	EU (*N* = 136)	Asia (*N* = 61)	ROW (*N* = 81)	North America (*N* = 51)	EU (*N* = 200)	Asia (*N* = 83)	ROW (*N* = 86)
n (%) *IR [95% CI, events per 100 PY]*								
TEAEs	33 (86.8) 369.17 [254.12–518.45]	75 (55.2) 125.25 [98.52–157.00]	47 (77.1) 309.42 [227.35–411.47]	43 (53.1) 102.28 [74.02–137.77]	39 (76.5) 343.08 [243.96–469.00]	103 (51.5) 139.47 [113.84–169.14]	63 (75.9) 337.90 [259.65–432.32]	43 (50.0) 98.64 [71.38–132.86]
Serious AEs	0 0.00 [0.00–15.07]	3 (2.2) 3.13 [0.65–9.15]	2 (3.3) 4.59 [0.56–16.57]	3 (3.7) 4.58 [0.94–13.39]	0 0.00 [0.00–13.15]	4 (2.0) 3.48 [0.95–8.91]	2 (2.4) 3.95 [0.48–14.28]	3 (3.5) 4.47 [0.92–13.08]
Severe AEs	1 (2.6) 4.12 [0.10–22.93]	2 (1.5) 2.09 [0.25–7.55]	0 0.00 [0.00–8.37]	4 (5.0) 6.11 [1.66–15.64]	1 (2.0) 3.59 [0.09–19.99]	3 (1.5) 2.61 [0.54–7.63]	0 0.00 [0.00–7.23]	4 (4.7) 5.97 [1.63–15.27]
Discontinuation due to AEs[b]	3 (7.9) 12.35 [2.55–36.10]	4 (2.9) 4.14 [1.13–10.60]	1 (6.4) 2.27 [0.06–12.67]	3 (3.7) 4.57 [0.94–13.37]	3 (5.9) 10.77 [2.22–31.46]	5 (2.5) 4.31 [1.40–10.06]	1 (1.2) 1.96 [0.05–10.93]	3 (3.5) 4.47 [0.92–13.06]

MedDRA version 23.0 coding dictionary applied. IRs (patients with events/100 PY), 95% CIs, and *n* (%) based a 28-day risk period (time of first to last study drug dose +28 days). For patients who switched from placebo to tofacitinib 5 mg BID after week 16, the first dose date refers to the date of the first tofacitinib dose. IRs for the 48-week cohorts are estimated without adjusting for study. Exact Poisson (adjusted for PY) 95% CI are provided for IRs.

[a]Within the 48-week cohorts, patients in the Phase 2 RCT received tofacitinib to week 12

[b]Discontinuation due to AEs is a mixture of discontinuation of study (Phase 2 RCT) and discontinuation of study treatment (Phase 3 RCT) due to AEs

AE, adverse event; AS, ankylosing spondylitis; BID, twice daily; CI, confidence interval; EU, European Union; IR, incidence rate; MedDRA, Medical Dictionary for

Regulatory Activities; *N*, total number of patients included in analysis; *n*, number of patients with event; PY, patient-years; RCT, randomized controlled trial; ROW, rest of the world; TEAE, treatment-emergent adverse event

Supplementary Table 8. Summary of all TEAEs, serious AEs, severe AEs, discontinuations due to AEs, and AEs of special interest for patients in the 48-week only-tofacitinib 5 mg BID and all-tofacitinib cohorts[a] (AS Phase 2/3 safety cohorts), by prior bDMARD history.

	48-week only-tofacitinib 5 mg BID Cohort		48-week all-tofacitinib cohort	
	bDMARD-naïve<br>(*N* = 258)	bDMARD-experienced[b]<br>(*N* = 58)	bDMARD-naïve<br>(*N* = 362)	bDMARD-experienced[b]<br>(*N* = 58)
n (%)<br>*IR [95% CI, events per 100 PY]*				
TEAEs	156 (60.5)<br>148.31 [125.95–173.49]	42 (72.4)<br>201.32 [145.09–272.12]	206 (56.9)<br>162.72 [141.26–186.52]	42 (72.4)<br>201.32 [145.09–272.12]
Serious AEs	6 (2.3)<br>3.23 [1.19–7.04]	2 (3.5)<br>4.55 [0.55–16.45]	7 (1.9)<br>3.23 [1.30–6.65]	2 (3.5)<br>4.55 [0.55–16.45]
Severe AEs	4 (1.6)<br>2.15 [0.59–5.51]	3 (5.2)<br>6.86 [1.41–20.05]	5 (1.4)<br>2.30 [0.75–5.37]	3 (5.2)<br>6.86 [1.41–20.05]
Discontinuation due to AEs[c]	5 (1.9)<br>2.68 [0.87–6.25]	6 (10.3)<br>13.74 [5.04–29.90]	6 (1.7)<br>2.75 [1.01–5.98]	6 (10.3)<br>13.74 [5.04–29.90]
AEs of special interest				
Serious infections[d]	1 (0.4)<br>0.53 [0.01–2.98]	0<br>0.00 [0.00–8.37]	1 (0.3)<br>0.46 [0.01–2.55]	0<br>0.00 [0.00–8.37]
Herpes zoster	3 (1.2)<br>1.61 [0.33–4.70]	2 (3.5)<br>4.64 [0.56–16.74]	5 (1.4)<br>2.30 [0.75–5.36]	2 (3.5)<br>4.64 [0.56–16.74]
Opportunistic infections[e]	0<br>0.00 [0.00–1.97]	0<br>0.00 [0.00, 8.37]	0<br>0.00 [0.00–1.69]	0<br>0.00 [0.00–8.37]
Tuberculosis[e]	0<br>0.00 [0.00–1.97]	0<br>0.00 [0.00–8.37]	0<br>0.00 [0.00–1.69]	0<br>0.00 [0.00–8.37]

MedDRA version 23.0 coding dictionary applied. IRs (patients with events/100 PY), 95% CIs, and *n* (%) based on a 28-day risk period (time of first to last study drug dose +28 days). For patients who switched from placebo to tofacitinib 5 mg BID after week 16, the first dose date refers to the date of the first tofacitinib dose. IRs for the 48-week cohorts are estimated without adjusting for study. Exact Poisson (adjusted for PY) 95% CI are provided for IRs

[a]Within the 48-week cohorts, patients in the Phase 2 RCT received tofacitinib to week 12

[b]Inadequate response to ≤ 2 TNFi or TNFi-experienced (non-inadequate response)

[c]Discontinuation due to AEs is a mixture of discontinuation of study (Phase 2 RCT) and discontinuation of study treatment (Phase 3 RCT) due to AEs

[d]Any event in the Infections and Infestations System Organ Class and classified as serious

[e]Adjudicated events in both AS Phase 2 and 3 RCTs

AE, adverse event; AS, ankylosing spondylitis; bDMARD, biologic disease-modifying antirheumatic drug; BID, twice daily; CI, confidence interval; IR, incidence rate; MedDRA, Medical Dictionary for Regulatory Activities; *N*, total number of patients included in analysis; *n*, number of patients with event; PY, patient-years; RCT, randomized controlled trial; TEAE, treatment-emergent adverse event; TNFi, tumor necrosis factor inhibitors

Supplementary Table 9. Summary of all TEAEs, serious AEs, severe AEs, and discontinuations due to AEs for patients in the 48-week only-tofacitinib 5 mg BID and all-tofacitinib cohorts[a] (AS Phase 2/3 safety cohorts), by Day 1 concomitant csDMARD use.

	48-week only-tofacitinib 5 mg BID cohort		48-week all-tofacitinib cohort	
	Yes<br>(*N* = 89)	No<br>(*N* = 227)	Yes<br>(*N* = 128)	No<br>(*N* = 292)
n (%)<br>*IR [95% CI, events per 100 PY]*				
TEAEs	52 (58.4)<br>145.11 [108.37–190.29]	146 (64.3)<br>161.84 [136.65–190.32]	74 (57.8)<br>169.76 [133.29–213.11]	174 (59.6)<br>167.52 [143.55–194.34]
Serious AEs	1 (1.1)<br>1.59 [0.04–8.87]	7 (3.1)<br>4.20 [1.69–8.66]	2 (1.6)<br>2.68 [0.32–9.67]	7 (2.4)<br>3.76 [1.51–7.76]
Severe AEs	2 (2.3)<br>3.22 [0.39–11.65]	5 (2.2)<br>2.99 [0.97–6.97]	2 (1.6)<br>2.70 [0.33–9.75]	6 (2.1)<br>3.21 [1.18–6.99]
Discontinuation due to AEs[b]	2 (2.3)<br>3.18 [0.39–11.50]	9 (4.0)<br>5.37 [2.45–10.19]	2 (1.6)<br>2.67 [0.32–9.65]	10 (3.4)<br>5.35 [2.56–9.83]

MedDRA version 23.0 coding dictionary applied. IRs (patients with events/100 PY), 95% CIs, and *n* (%) based on a 28-day risk period (time of first to last study drug dose +28 days). For patients who switched from placebo to tofacitinib 5 mg BID after week 16, the first dose date refers to the date of the first tofacitinib dose. IRs for the 48-week cohorts are estimated without adjusting for study. Exact Poisson (adjusted for PY) 95% CI are provided for IRs

[a]Within the 48-week cohorts, patients in the Phase 2 RCT received tofacitinib to week 12

[b]Discontinuation due to AEs is a mixture of discontinuation of study (Phase 2 RCT) and discontinuation of study treatment (Phase 3 RCT) due to AEs

AE, adverse event; AS, ankylosing spondylitis; BID, twice daily; CI, confidence interval; csDMARD, conventional synthetic disease-modifying antirheumatic drug; IR, incidence rate; MedDRA, Medical Dictionary for Regulatory Activities; *N*, total number of patients included in analysis; *n*, number of patients with event; PY, patient-years; RCT, randomized controlled trial; TEAE, treatment-emergent adverse event

Supplementary Table 10 AEs of special interest for patients in the AS real-world cohort[a]

	N = 2253	
	Censored at 16 weeks	Censored at 48 weeks
n (%)<br>*IR [95% CI, events per 100 PY]*		
Serious infections[b,c]	24 (1.1)<br>3.24 [2.00–4.98]	47 (2.1)<br>3.59 [2.57–4.87]
Opportunistic infections (excluding tuberculosis and herpes zoster)[b]	2 (0.1)<br>0.24 [0.03–0.91]	3 (0.1)<br>0.17 [0.03–0.51]
Tuberculosis[b]	0<br>0.00 [0.00–0.61]	0<br>0.00 [0.00–0.31]
Herpes zoster[b,d]	5 (0.2)<br>0.53 [0.17–1.26]	12 (0.5)<br>0.74 [0.37–1.34]
Malignancy, excluding NMSC[e,f]	0<br>0.00 [0.00–0.57]	3 (0.1)[g]<br>0.15 [0.03–0.43]
NMSC[e]	3 (0.1)<br>0.32 [0.06–0.95]	6 (0.3)<br>0.29 [0.10–0.64]
MACE[g,h]	5 (0.2)<br>0.60 [0.19–1.43]	10 (0.4)<br>0.61 [0.28–1.16]
VTE[g,i]	5 (0.2)<br>1.04 [0.32–2.49]	7 (0.3)<br>0.62 [0.23–1.33]
DVT[g]	4 (0.2)<br>0.80 [0.20–2.15]	5 (0.2)<br>0.39 [0.11–0.96]
PE[g]	1 (< 0.1)<br>0.23 [0.01–1.29]	2 (0.1)<br>0.22 [0.03–0.80]
ATE[g]	0<br>0.00 [0.00–0.57]	0<br>0.00 [0.00–0.26]
GI perforation[g]	0<br>0.00 [0.00–0.57]	0<br>0.00 [0.00–0.26]
Interstitial lung disease[e]	0<br>0.00 [0.00–0.57]	2 (0.1)<br>0.10 [0.01–0.37]

Crude IRs (patients with events/100 PY) and 95% CIs were age- and sex-weighted per the AS Phase 3 RCT. IRs are for initiation of any bDMARD ever for bDMARD-naïve patients and for initiation of a new bDMARD during the study period for the bDMARD-experienced patients

[a]Exclusion criteria similar to those within the tofacitinib AS Phase 3 RCT were applied. Patients were bDMARD initiators who were bDMARD-naïve or -experienced as of index date, with data censored at 16 or 48 weeks for the first initiation

[b]Within 30 days of discontinuation of exposure to drug

[c]Defined as infections (viral, bacterial, and fungal) resulting in hospitalization or use of parenteral antibiotics

[d]Includes serious and non-serious herpes zoster events

[e]Ever exposure to drug

[f]One case each of breast cancer, prostate cancer, and lymphoma

[g]Within 90 days of discontinuation of exposure to drug

[h]Includes nonfatal myocardial infarction, nonfatal stroke, or death from cardiovascular causes

[I]Includes PE or DVT

AE, adverse event; AS, ankylosing spondylitis; ATE, arterial thromboembolism; bDMARD, biologic disease-modifying antirheumatic drug; CI, confidence interval; DVT, deep vein thrombosis; GI, gastrointestinal; IR, incidence rate; MACE, major adverse cardiovascular events; *N*, total number of patients included in analysis; *n*, number of patients with event; NMSC, nonmelanoma skin cancer; PE, pulmonary embolism; PY, patient-years; RCT, randomized controlled trial; VTE, venous thromboembolism

Supplementary Figure 1. Mean absolute (±SE) values for **a** AST, **b** ALT, and **c** total bilirubin for patients with AS in the 16-week placebo-controlled cohort and the 48-week only-tofacitinib 5 mg BID and all-tofacitinib cohorts (AS Phase 2/3 safety cohorts)[a]

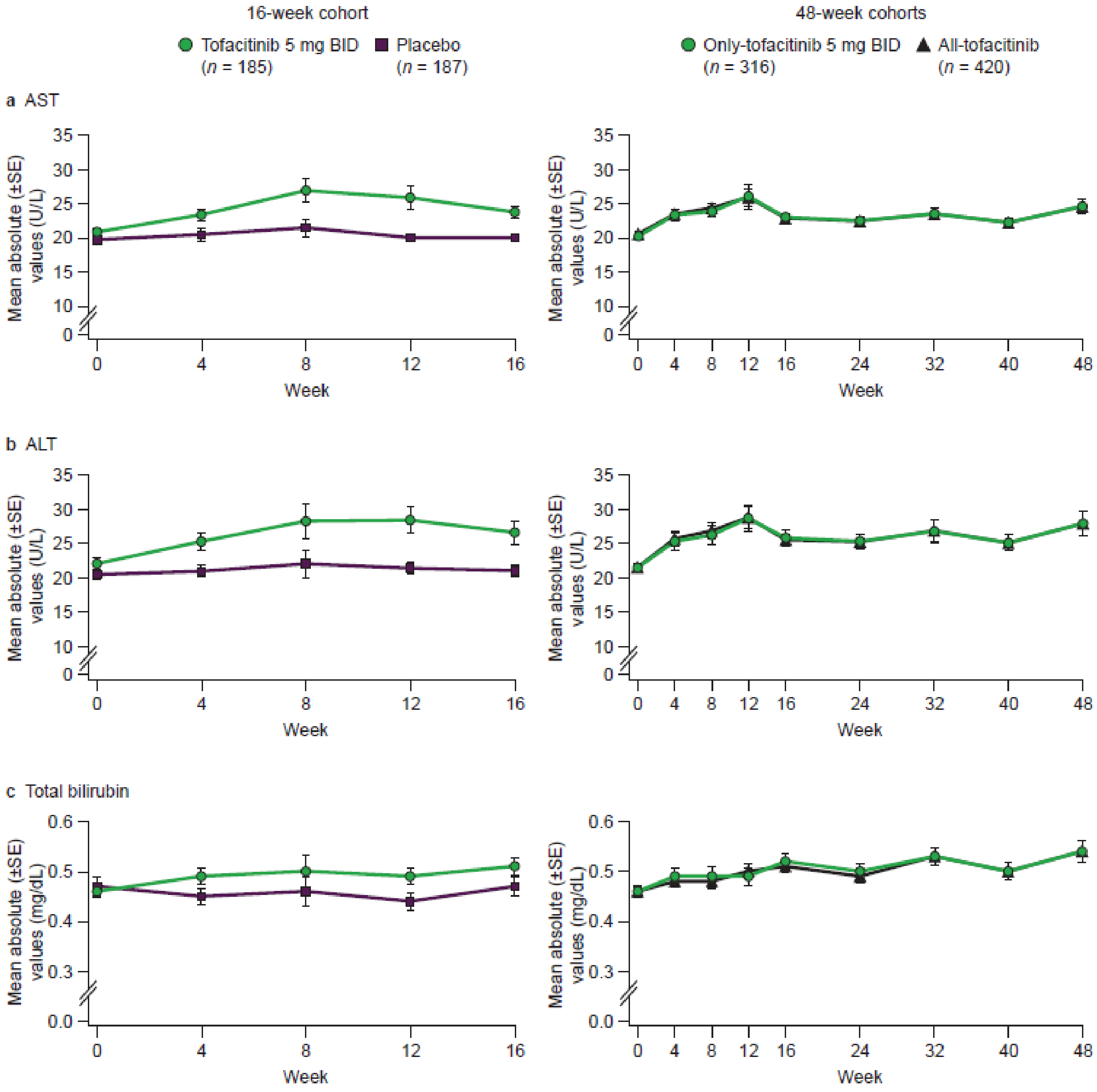

[a]Within the 16-week placebo-controlled and 48-week cohorts, patients in the Phase 2 RCT received tofacitinib to week 12

ALT, alanine aminotransferase; AS, ankylosing spondylitis; AST, aspartate aminotransferase; BID, twice daily; *N*, total number of patients; RCT, randomized controlled trial; SE, standard error

Supplementary Table 11. Incidence of laboratory values meeting protocol criteria for monitoring and discontinuation of study drug for patients in the 16-week placebo-controlled cohort and the 48-week only-tofacitinib 5 mg BID and all-tofacitinib cohorts (AS Phase 2/3 safety cohorts)[a]

	16-week placebo-controlled cohort		48-week cohort	
	Tofacitinib 5 mg BID ($N = 185$)	Placebo ($N = 187$)	Only-tofacitinib 5 mg BID ($N = 316$)	All-tofacitinib ($N = 420$)
Monitoring criteria, *n* (%)				
Met any monitoring criteria	4 (2.2)	9 (4.8)	17 (5.4)	20 (4.8)
Any single hemoglobin drops > 2 g/dL below baseline	0	4 (2.1)	6 (1.9)	6 (1.4)
Absolute neutrophil count < 1.2 × 10^9/L	1 (0.5)	0	1 (0.3)	2 (0.5)
Absolute lymphocyte count < 0.5 × 10^9/L	1 (0.5)	0	1 (0.3)	1 (0.2)
Platelet count < 100 × 10^9/L	0	1 (0.5)	0	0
Serum creatinine increase > 50% or increase > 0.5 mg/dL over the average of screening and baseline values	0	1 (0.5)	7 (2.2)	8 (1.9)
Any creatine kinase > 5 × ULN	2 (1.1)	3 (1.6)	2 (0.6)	3 (0.7)

Discontinuation criteria, *n* (%)				
Met any discontinuation criteria	1 (0.5)	1 (0.5)	2 (0.6)	2 (0.5)
Two sequential hemoglobin < 8.0 g/dL or decreases of > 30% from baseline value	0	0	0	0
Two sequential absolute neutrophil counts < 1.0 × 10^9/L	0	0	0	0
Two sequential absolute lymphocyte count < 500 lymphocytes/mm^3	0	0	0	0
Two sequential platelet counts < 75 × 10^9/L	0	0	0	0
Two sequential AST or ALT elevations ≥ 3 × ULN with at least one total bilirubin ≥ 2 × ULN	0	0	0	0
Two sequential AST or ALT elevations ≥ 3 × ULN accompanied by hepatic injury (e.g., new-onset elevated PT/INR)	0	0	0	0
Two sequential AST or ALT elevations > 5 × ULN	1 (0.5)	0	2 (0.6)	2 (0.5)
Two sequential serum creatinine increase > 50% and increase > 0.5 mg/dL over the average of screening and baseline values	0	0	0	0
Two sequential creatine kinase elevations > 10 × ULN	0	0	0	0
A confirmed positive urine pregnancy test in a woman of childbearing potential	0	1 (0.5)	0	0

A patient may have met multiple criteria. Each patient was counted once for each row.

Baseline was defined as the last non-missing assessment prior to the first dose of investigational product (including placebo) in the 16-week placebo-controlled cohorts and

prior to the first dose of tofacitinib in the 48-week cohorts

[a]Within the 16-week placebo-controlled and 48-week cohorts, patients in the Phase 2 RCT received tofacitinib to week 12

ALT, alanine aminotransferase; AS, ankylosing spondylitis; AST, aspartate aminotransferase; BID, twice daily; INR, international normalized ratio; *N*, total number of patients included in analysis; *n*, number of patients who met the criteria; PT, prothrombin time; RCT, randomized controlled trial; ULN, upper limit of normal

Supplementary Table 12. Confirmed[a] transaminase and bilirubin elevations for patients in the 16-week placebo-controlled cohort and the 48-week only-tofacitinib 5 mg BID and all-tofacitinib cohorts (AS Phase 2/3 safety cohorts)[b]

	16-week placebo-controlled cohort		48-week cohort	
	Tofacitinib 5 mg BID (*N* = 185; *N*1 = 184)	Placebo (*N* = 187; *N*1 = 184)	Only-tofacitinib 5 mg BID (*N* = 316; *N*1 = 314)	All-tofacitinib (*N* = 420; *N*1 = 415)
ALT, *n* (%)				
≥ 2 × ULN	4 (2.2)	1 (0.5)	7 (2.2)	8 (1.9)
≥ 3 × ULN	1 (0.5)	0	2 (0.6)	2 (0.5)
≥ 5 × ULN	1 (0.5)	0	2 (0.6)	2 (0.5)
≥ 10 × ULN	0	0	0	0
AST, *n* (%)				
≥ 2 × ULN	3 (1.6)	0	6 (1.9)	6 (1.4)
≥ 3 × ULN	1 (0.5)	0	1 (0.3)	1 (0.2)
≥ 5 × ULN	0	0	0	0
≥ 10 × ULN	0	0	0	0
Bilirubin, *n* (%)				
≥ 2 × ULN	0	0	0	0
≥ 3 × ULN	0	0	0	0

[a]At least two consecutive measurements within the patient. [b]Within the 16-week placebo-controlled and 48-week cohorts, patients in the Phase 2 RCT received tofacitinib to week 12ALT, alanine aminotransferase; AS, ankylosing spondylitis; AST, aspartate aminotransferase; BID, twice daily; *N*, total number of patients included in analysis; *N*1, number of patients with a post-baseline observation; *n*, number of patients who met the criteria; RCT, randomized controlled trial; ULN, upper limit of normal.

Supplementary Figure 2. Mean absolute (±SE) values for **a** hemoglobin, **b** neutrophils, **c** lymphocytes, and **d** platelets for patients in the 16-week placebo-controlled cohort and the 48-week only-tofacitinib 5 mg BID and all-tofacitinib cohorts (AS Phase 2/3 safety cohorts)[a]

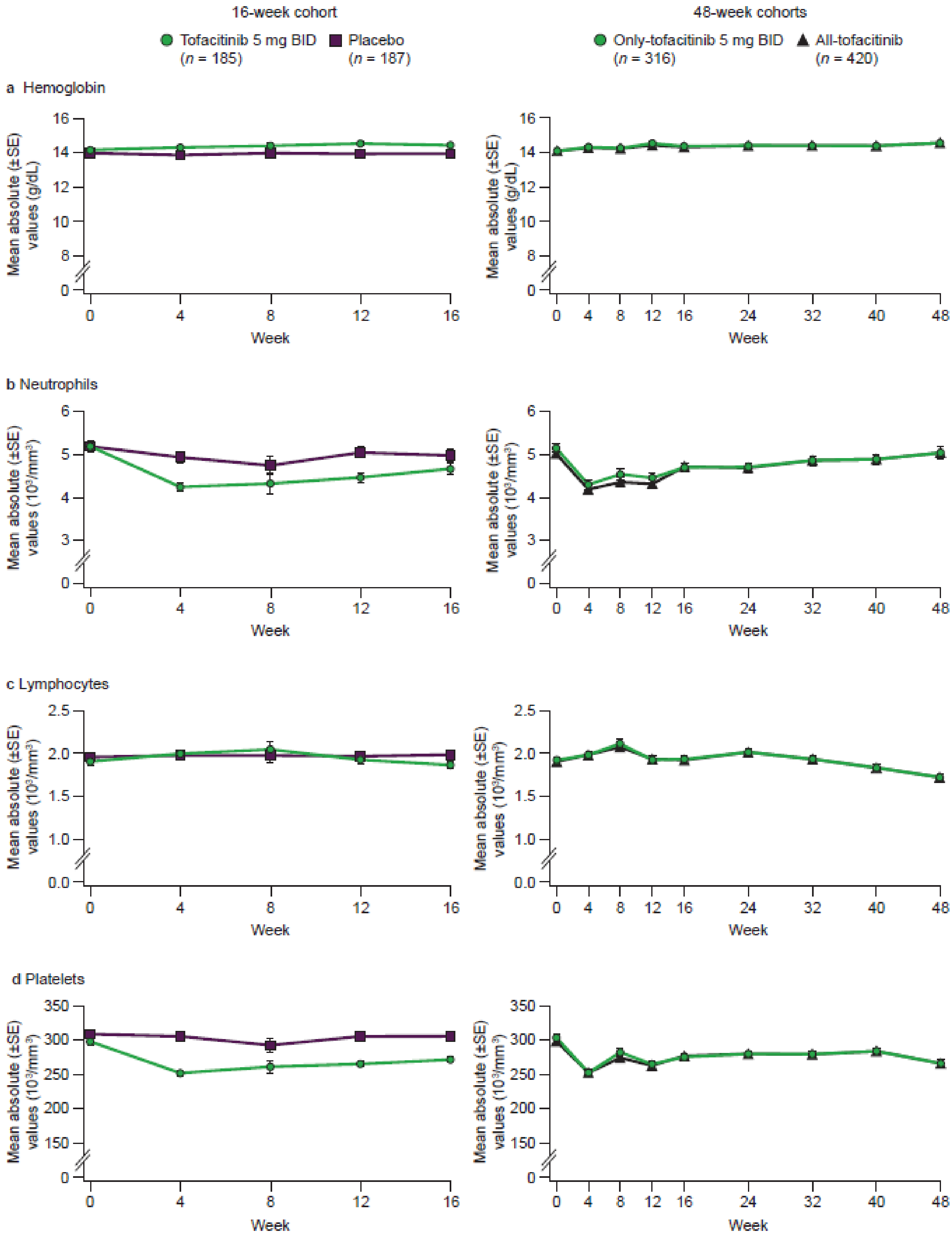

[a]Within the 16-week placebo-controlled and 48-week cohorts, patients in the Phase 2 RCT received tofacitinib to week 12

AS, ankylosing spondylitis; BID, twice daily; *N*, total number of patients; RCT, randomized controlled trial; SE, standard error.

Supplementary Figure 3. Mean (±SE) change from baseline[a] in **a** hemoglobin, **b** absolute neutrophil count, **c** absolute lymphocyte count, and **d** platelet count for patients in the 16-week placebo-controlled cohort and the 48-week only-tofacitinib 5 mg BID and all-tofacitinib cohorts (AS Phase 2/3 clinical trial cohorts)[b]

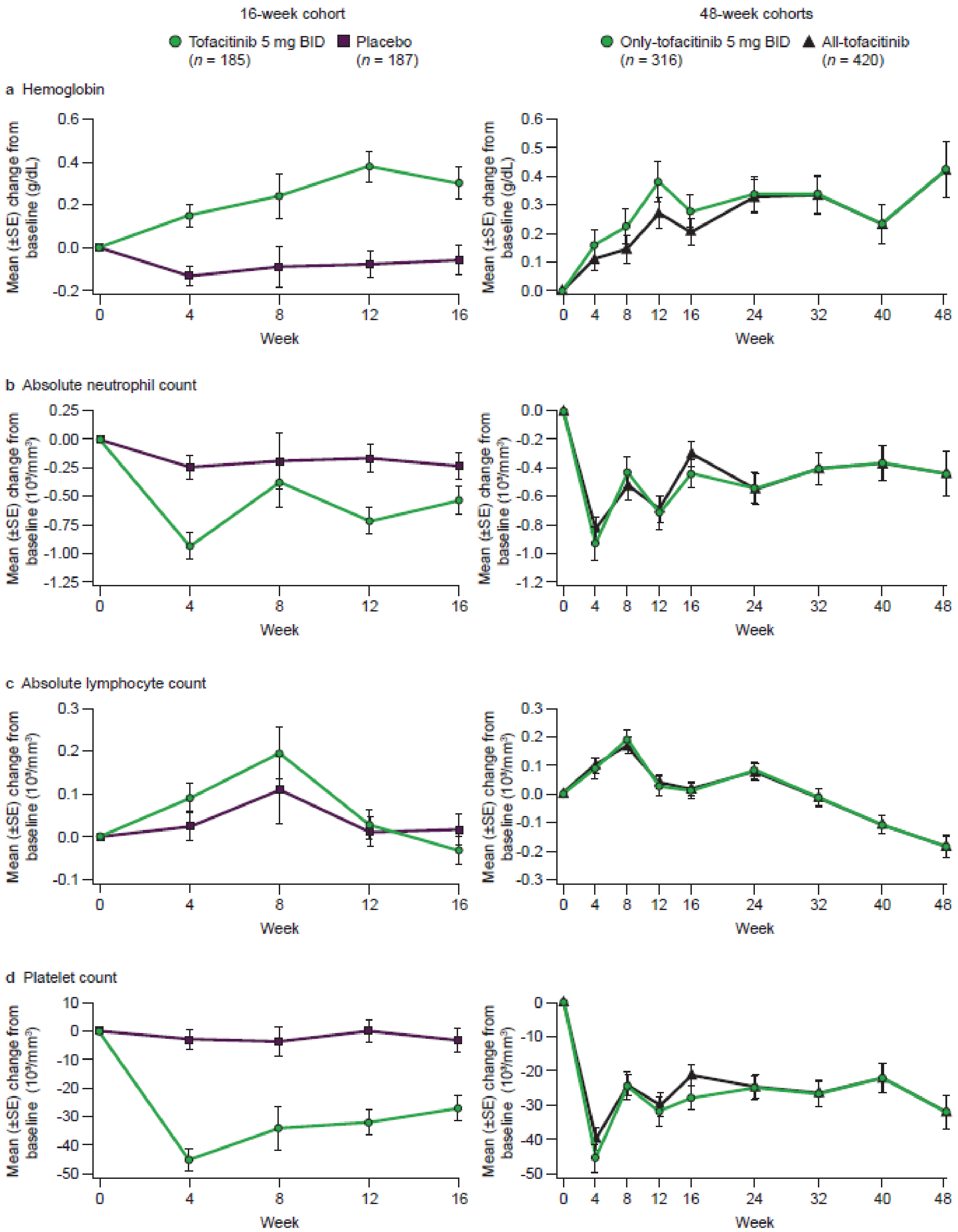

[a]Baseline was defined as the last non-missing assessment prior to the first dose of investigational product (including placebo) in the 16-week placebo-controlled cohort and prior to the first dose of tofacitinib in the 48-week cohorts. [b]Within the 16-week placebo-controlled 48-week cohorts, patients in the Phase 2 RCT received tofacitinib to week 12. AS, ankylosing spondylitis; BID, twice daily; *N*, total number of patients; RCT, randomized controlled trial; SE, standard error

Supplementary Figure 4. Mean absolute (±SE) values for **a** total cholesterol, **b** HDL-c, **c** LDL-c, and **d** triglycerides for patients in the 16-week placebo-controlled cohort and the 48-week only-tofacitinib 5 mg BID and all-tofacitinib cohorts (AS Phase 2/3 safety cohorts)[a]

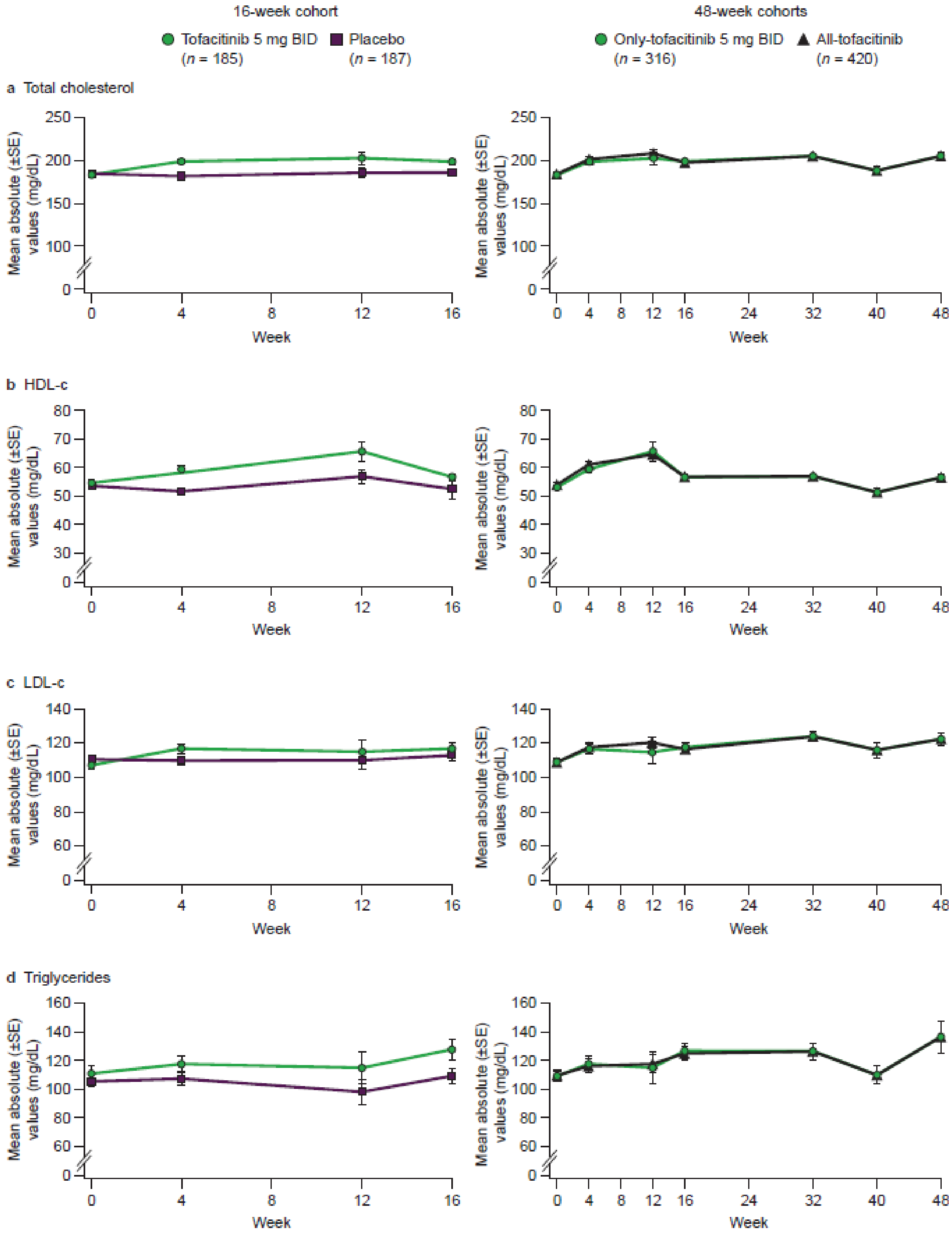

[a]Within the 16-week placebo-controlled and 48-week cohorts, patients in the Phase 2 RCT received tofacitinib to week 12. AS, ankylosing spondylitis; BID, twice daily; HDL-c, high-density lipoprotein-cholesterol; LDL-c, low-density lipoprotein-cholesterol; *N*, total number of patients; RCT, randomized controlled trial; SE, standard error.

Supplementary Figure 5. Mean absolute (±SE) values for **a** creatine kinase and **b** serum creatinine for patients in the 16-week placebo-controlled cohort and the 48-week only-tofacitinib 5 mg BID and all-tofacitinib cohorts (AS Phase 2/3 safety cohorts)[a]

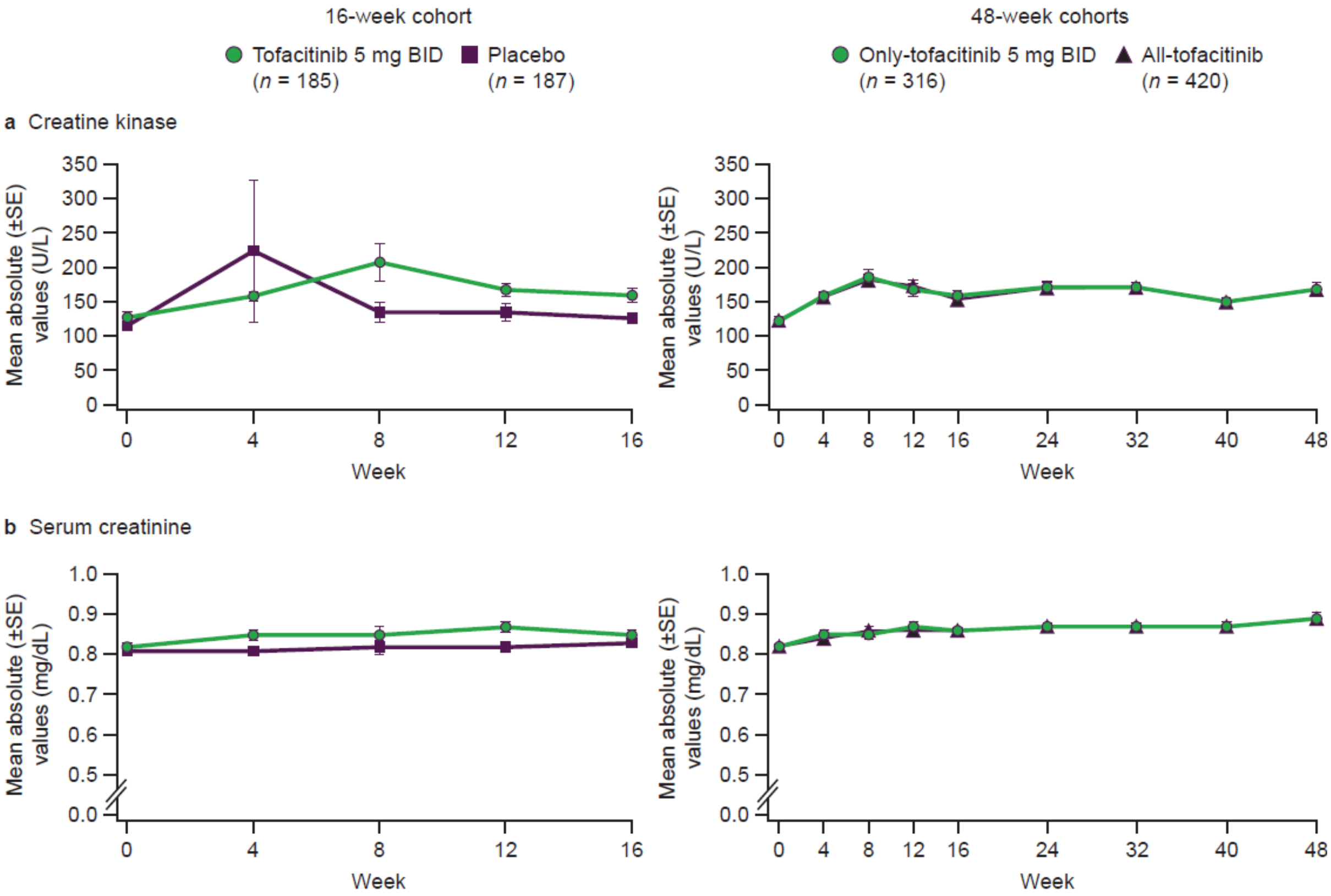

[a]Within the 16-week placebo-controlled 48-week cohorts, patients in the Phase 2 RCT received tofacitinib to week 12

AS, ankylosing spondylitis; BID, twice daily; *N*, total number of patients; RCT, randomized controlled trial; SE, standard error

Supplementary Figure 6. Mean (±SE) change from baseline[a] in **a** creatine kinase and **b** serum creatinine for patients in the 16-week placebo-controlled cohort and the 48-week only-tofacitinib 5 mg BID and all-tofacitinib cohorts (AS Phase 2/3 safety cohorts)[b]

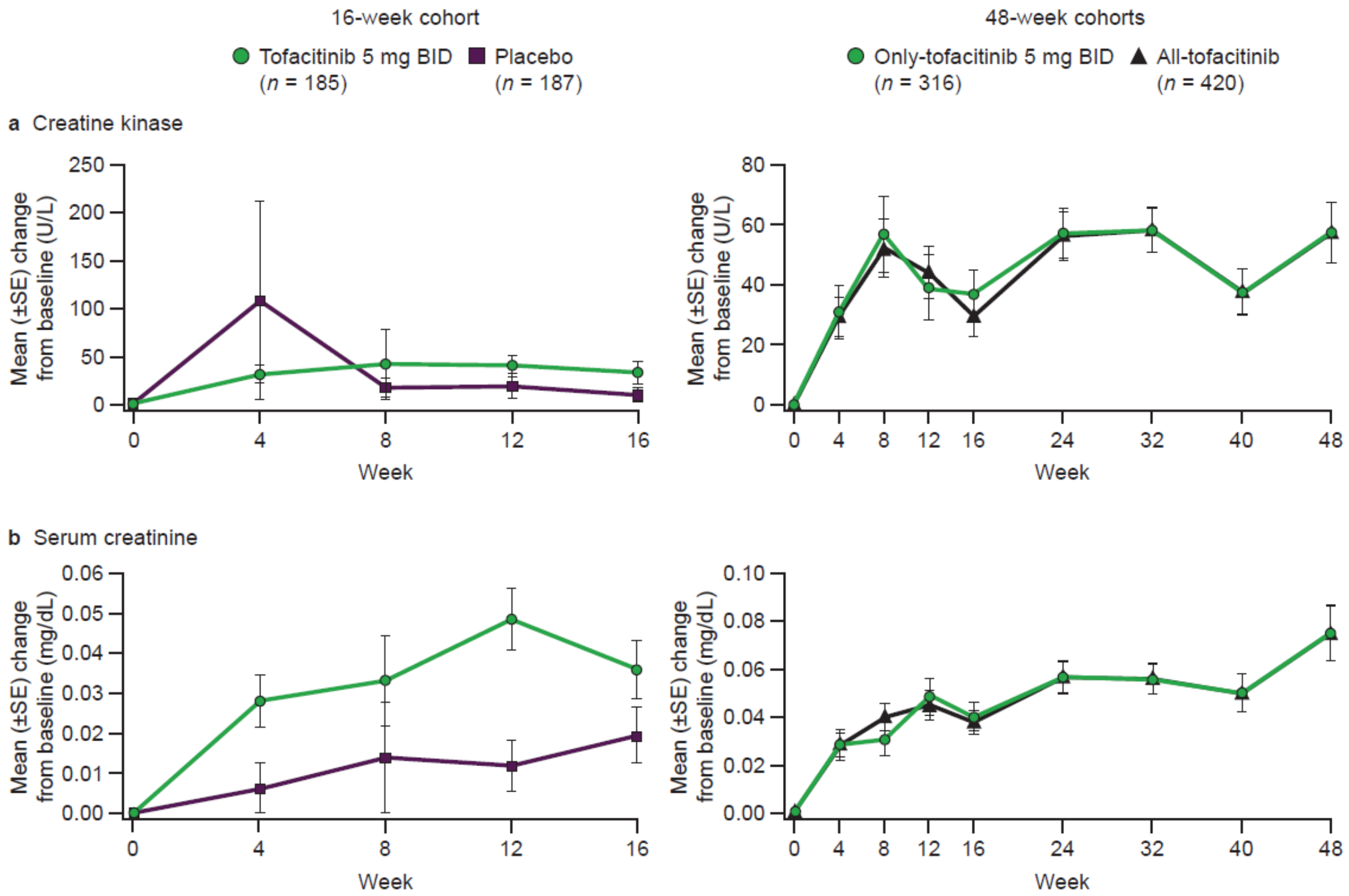

[a]Baseline was defined as the last non-missing assessment prior to the first dose of investigational product (including placebo) in the 16-week placebo-controlled cohorts and prior to the first dose of tofacitinib in the 48-week cohorts

[b]Within the 16-week placebo-controlled and 48-week cohorts, patients in the Phase 2 RCT received tofacitinib to week 12

AS, ankylosing spondylitis; BID, twice daily; *N*, total number of patients; RCT, randomized controlled trial; SE, standard error

Supplementary Figure 7. Mean (±SE) change from baseline[a] in **a** systolic and **b** diastolic blood pressure for patients in the 16-week placebo-controlled cohort and the 48-week only-tofacitinib 5 mg BID and all-tofacitinib cohorts (AS Phase 2/3 safety cohorts)[b]

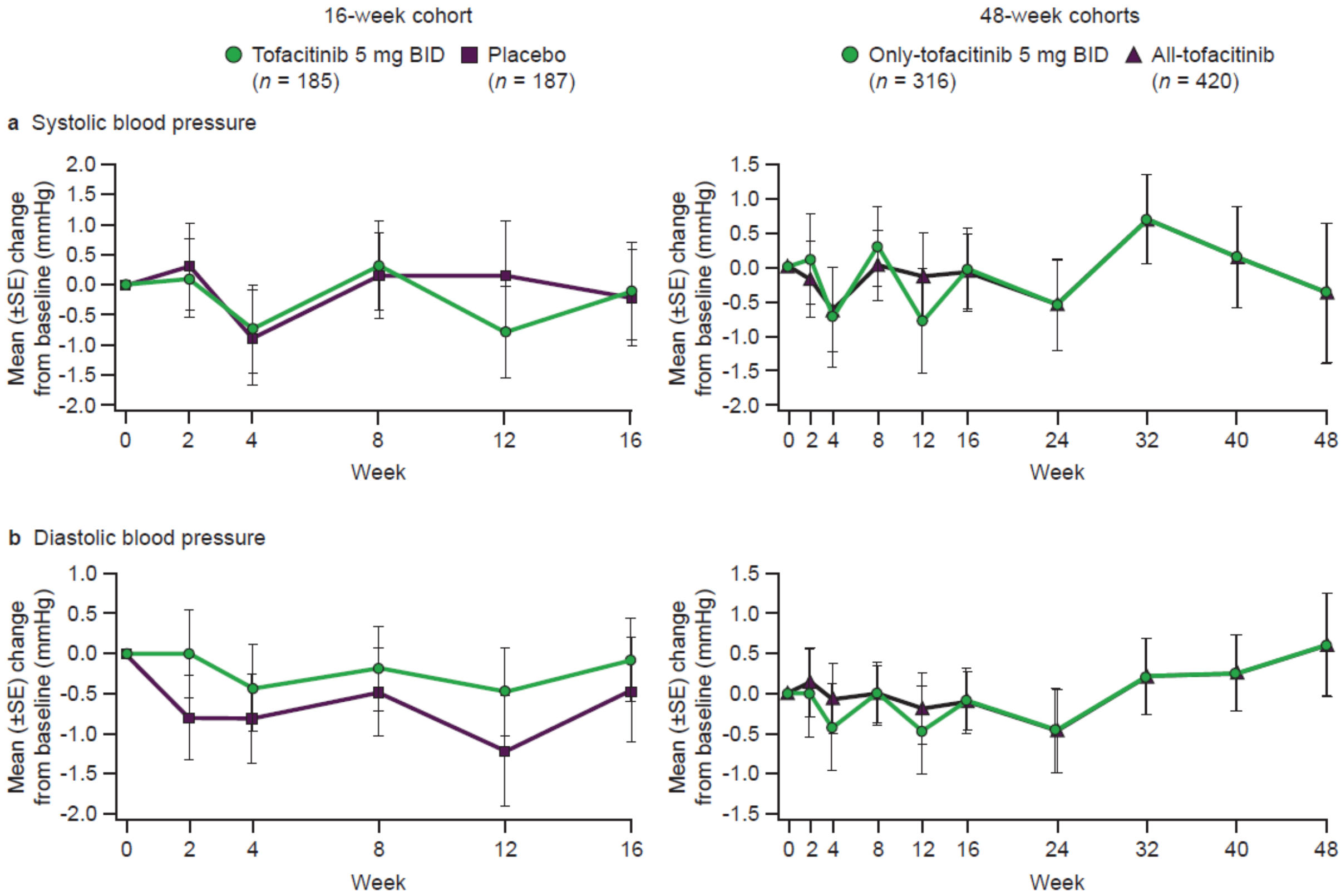

[a]Baseline was defined as the last non-missing assessment prior to the first dose of investigational product (including placebo) in the 16-week placebo-controlled cohorts and prior to the first dose of tofacitinib in the 48-week cohorts

[b]Within the 16-week placebo-controlled and 48-week cohorts, patients in the Phase 2 RCT received tofacitinib to week 12

AS, ankylosing spondylitis; BID, twice daily; *N*, total number of patients; RCT, randomized controlled trial; SE, standard error

Supplementary Figure 8. Mean (±SE) change from baseline[a] in weight (kg) for patients in the 16-week placebo-controlled cohort and the 48-week only-tofacitinib 5 mg BID and all-tofacitinib cohorts (AS Phase 2/3 safety cohorts)[b]

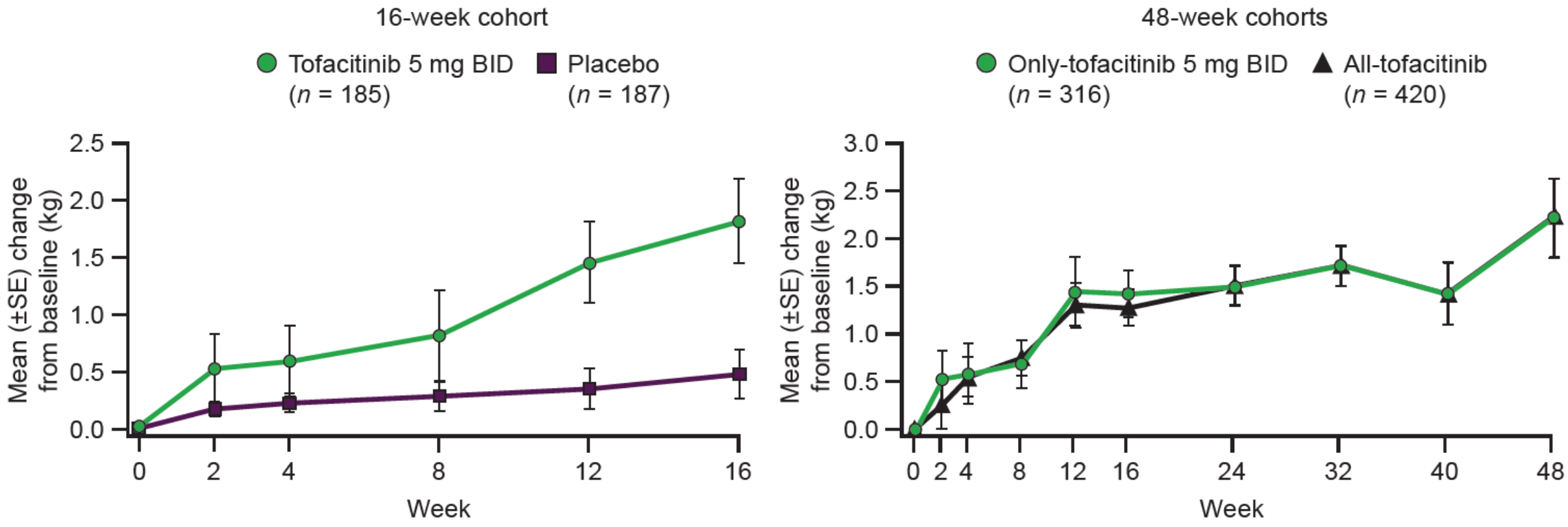

[a]Baseline was defined as the last non-missing assessment prior to the first dose of investigational product (including placebo) in the 16-week placebo-controlled cohorts and prior to the first dose of tofacitinib in the 48-week cohorts

[b]Within the 16-week placebo-controlled and 48-week cohorts, patients in the Phase 2 RCT received tofacitinib to week 12

AS, ankylosing spondylitis; BID, twice daily; *N*, total number of patients; RCT, randomized controlled trial; SE, standard error.
